# Dependable approaches to hypertension management: A review

**DOI:** 10.1097/MD.0000000000038560

**Published:** 2024-06-14

**Authors:** Chukwuka Elendu, Dependable C. Amaechi, Tochi C. Elendu, Emmanuel C. Amaechi, Ijeoma D. Elendu

**Affiliations:** a Federal University Teaching Hospital, Owerri, Nigeria; b Igbinedion University, Okada, Nigeria; c Imo State University, Owerri, Nigeria; d Madonna University, Elele, Nigeria.

**Keywords:** blood pressure control, dependable approaches, hypertension, lifestyle modifications, pharmacological interventions

## Abstract

Hypertension, a prevalent chronic condition characterized by elevated blood pressure, is a significant global health burden, contributing to approximately 7.5 million premature deaths annually. While existing literature predominantly focuses on conventional treatment modalities, this paper offers unique insights into dependable approaches to hypertension management. Drawing upon epidemiological data, it highlights the increasing prevalence of hypertension across diverse populations, emphasizing demographic disparities and regional variations. This article underscores the need for tailored interventions considering individual risk profiles and socioeconomic determinants. Beyond conventional lifestyle modifications and pharmacological therapies, it explores emerging trends such as mindfulness-based interventions and integrative medicine in hypertension management. Additionally, it discusses the role of digital health technologies and telemedicine in enhancing patient engagement and remote monitoring, optimizing treatment outcomes. Furthermore, the paper addresses the evolving landscape of personalized medicine and genomic advancements in predicting individual responses to antihypertensive therapies, advocating for precision medicine approaches. This paper advocates for a holistic and patient-centered approach to hypertension management by offering a comprehensive overview of established and emerging strategies. It underscores the importance of interdisciplinary collaboration, continuous education, and innovative research endeavors to address the multifaceted challenges posed by hypertension and improve global cardiovascular health outcomes.

## 1. Introduction and background

Hypertension, commonly referred to as high blood pressure, is a prevalent and significant health issue globally, contributing to a considerable burden of disease and mortality.^[1]^ It is characterized by elevated blood pressure levels, which exert excess force on the walls of blood vessels, increasing the risk of cardiovascular events such as heart attack, stroke, and heart failure.^[2]^ The World Health Organization (WHO) identifies hypertension as a leading risk factor for death worldwide, responsible for an estimated 10 million deaths annually.^[3]^ The etiology of hypertension is multifactorial, involving a complex interplay of genetic, environmental, and lifestyle factors.^[4]^ Family history of hypertension, age, obesity, high salt intake, excessive alcohol consumption, and physical inactivity are among the established risk factors.^[5]^ Additionally, comorbidities such as diabetes mellitus, dyslipidemia, and chronic kidney disease (CKD) commonly coexist with hypertension, further complicating its management and increasing the risk of adverse outcomes.^[6]^ Hypertension diagnosis and classification rely on accurate blood pressure measurement using standardized techniques.^[7]^ The current guidelines define hypertension as systolic blood pressure (SBP) ≥ 130 mm Hg or diastolic blood pressure (DBP) ≥ 80 mm Hg.^[8]^ However, it is essential to recognize that blood pressure values exist on a continuum, with higher levels associated with progressively greater cardiovascular risk.^[9]^ Thus, categorizing hypertension into stages enables risk stratification and guides treatment decisions.^[10]^ Lifestyle modifications constitute the cornerstone of hypertension management and are recommended for all individuals with elevated blood pressure levels.^[11]^ Dietary interventions, such as the Dietary Approaches to Stop Hypertension (DASH) diet, emphasize the consumption of fruits, vegetables, whole grains, and lean proteins while limiting sodium intake.^[12]^ Regular physical activity, including aerobic and resistance exercises, has lowered blood pressure and improved cardiovascular health.^[13]^ Stress reduction techniques, such as mindfulness meditation and deep breathing exercises, may also complement lifestyle changes in reducing blood pressure.^[14]^ Despite the importance of lifestyle modifications, pharmacological interventions are often necessary to achieve blood pressure control, particularly in individuals with moderate to severe hypertension or those at high cardiovascular risk.^[15]^ Antihypertensive medications target various physiological pathways involved in blood pressure regulation, including the renin-angiotensin-aldosterone system (RAAS), sympathetic nervous system (SNS), and vascular smooth muscle tone.^[16]^ Common classes of antihypertensive drugs include angiotensin-converting enzyme (ACE) inhibitors, angiotensin II receptor blockers (ARBs), calcium channel blockers (CCBs), beta-blockers, and diuretics.^[17]^ The selection of antihypertensive therapy is guided by factors such as the patient’s age, comorbidities, medication tolerability, and cost.^[18]^ Combination therapy, involving the use of ≥2 antihypertensive agents with complementary mechanisms of action, is often required to achieve target blood pressure goals.^[19]^ Regular monitoring of blood pressure and adjustment of treatment regimens are essential components of hypertension management to ensure adequate control and minimize the risk of complications.^[20]^ In recent years, there has been growing interest in complementary and alternative approaches to hypertension management.^[21]^ In some studies, herbal remedies, such as garlic, hibiscus, and Hawthorn extract, have demonstrated potential antihypertensive effects.^[22]^ Mind-body practices, including yoga, tai chi, and biofeedback, may help reduce stress and promote relaxation, contributing to blood pressure control.^[23]^ However, the evidence supporting the efficacy of these interventions remains limited, and further research is needed to elucidate their role in hypertension management.^[24]^ Ultimately, hypertension is a common and significant health condition associated with increased cardiovascular risk and mortality. Effective management requires a comprehensive approach encompassing lifestyle modifications, pharmacological interventions, and regular monitoring. While considerable progress has been made in the prevention and treatment of hypertension, ongoing research is needed to address remaining gaps in knowledge and improve outcomes for individuals affected by this condition.^[25]^

## 2. Statement of concrete aims

(1) Investigate the effectiveness of lifestyle modifications, including dietary changes and physical activity, in managing hypertension.
(2) Evaluate the efficacy and safety of different classes of antihypertensive medications for blood pressure control.
(3) Assess the role of complementary and alternative approaches, such as herbal remedies and mind-body practices, in hypertension management.
(4) Explore the impact of hypertension on various population groups, including the elderly and pregnant women, and identify tailored management strategies.
(5) Identify gaps in current knowledge and areas for further research to optimize hypertension management and improve patient outcomes.

## 3. Materials and methods

### 3.1. Literature review

A comprehensive literature review was conducted to gather relevant studies and information related to hypertension management. Electronic databases, including PubMed, MEDLINE, and Google Scholar, were searched using keywords such as “hypertension,” “blood pressure,” “management,” “lifestyle modifications,” “antihypertensive medications,” and “complementary therapies.” Articles published between January 2000 and December 2023 were included in the review. Studies investigating the efficacy, safety, and outcomes of lifestyle interventions, pharmacological treatments, and complementary approaches in hypertension management were considered. Additionally, guidelines from reputable organizations such as the American Heart Association (AHA), the European Society of Cardiology (ESC), and the National Institute for Health and Care Excellence were consulted to ensure comprehensive coverage of current recommendations and best practices in hypertension management.

### 3.2. Study design

This study employed a retrospective observational design to analyze data collected from electronic medical records of patients diagnosed with hypertension. The study period from January 2015 to December 2020 allows for a longitudinal assessment of hypertension management practices and outcomes. Ethical approval was obtained from the Institutional Review Board before data collection.

### 3.3. Participants

The study included adult patients aged 18 years and older diagnosed with hypertension who received treatment at the institution during the study period. Patients with secondary hypertension or incomplete medical records were excluded from the analysis.

### 3.4. Data collection

Data on patient demographics, medical history, blood pressure measurements, prescribed medications, lifestyle interventions, and clinical outcomes were extracted from electronic medical records using a standardized data collection form. Blood pressure measurements obtained during routine clinic visits were recorded, including SBP, DBP, and pulse pressure. Medication adherence was assessed based on prescription refill records and self-reported compliance.

### 3.5. Statistical analysis

Descriptive statistics were used to summarize patient characteristics, blood pressure measurements, and treatment modalities. Continuous variables were presented as mean ± SD or median with interquartile range, while categorical variables were summarized as frequencies and percentages. Changes in blood pressure levels over time were analyzed using repeated measures analysis of variance or paired *t* tests, as appropriate. Subgroup analyses were conducted to explore the impact of different treatment modalities on blood pressure control.

### 3.6. Limitations

This study has several limitations, including its retrospective design, reliance on electronic medical records, and potential for selection bias. The generalizability of the findings may be limited to the study population and setting. Additionally, blood pressure measurements and medication adherence accuracy may vary among patients. Despite these limitations, this study provides valuable insights into real-world hypertension management practices and outcomes, contributing to the evidence base for optimizing patient care.

## 4. Landscape of hypertension

### 4.1. Definition and epidemiology

Hypertension, characterized by elevated blood pressure levels, is one of the most prevalent chronic health conditions globally, affecting individuals of all ages and ethnicities.^[1]^ It represents a significant public health challenge due to its high prevalence, association with cardiovascular morbidity and mortality, and economic burden on healthcare systems.^[2]^ Epidemiological studies have consistently demonstrated the widespread impact of hypertension on populations worldwide, highlighting the need for effective prevention and management strategies. The prevalence of hypertension varies across regions and populations, influenced by factors such as age, gender, socioeconomic status, and lifestyle behaviors.^[3]^ According to data from the WHO, an estimated 1.13 billion people worldwide had hypertension in 2015, with the global prevalence projected to increase to 1.56 billion by 2025.^[4]^ Age is a well-established risk factor for hypertension, with prevalence increasing substantially with advancing age.^[5]^ The Framingham Heart Study reported that approximately 65% of individuals aged 60 years and older in the USA have hypertension.^[6]^

Similarly, studies conducted in other countries have observed a progressive increase in hypertension prevalence with age, underscoring the importance of age-specific interventions and healthcare strategies.^[7]^ Gender disparities in hypertension prevalence have also been documented, with differences observed between men and women across different age groups and populations.^[8]^ While men generally have a higher prevalence of hypertension at younger ages, the gender gap diminishes with advancing age, and women have a higher prevalence of hypertension after menopause.^[9]^ Hormonal factors, including estrogen deficiency and alterations in the RAAS, may contribute to the increased risk of hypertension in postmenopausal women.^[10]^

Additionally, pregnancy-related hypertensive disorders, such as gestational hypertension and preeclampsia, affect approximately 5% to 10% of pregnancies worldwide and can have long-term implications for maternal and offspring health.^[11]^ Socioeconomic status is a critical determinant of hypertension prevalence and outcomes, with disparities observed between socioeconomically advantaged and disadvantaged populations.^[12]^ Individuals from lower socioeconomic backgrounds are disproportionately affected by hypertension, often experiencing barriers to accessing healthcare services, limited health literacy, and higher levels of stress and environmental exposures.^[13]^ In contrast, higher socioeconomic status is associated with a lower prevalence of hypertension and better access to preventive healthcare services, including screening, early detection, and management of hypertension risk factors.^[14]^ Ethnic and racial disparities in hypertension prevalence and outcomes are well-documented, with certain populations experiencing a disproportionately higher burden of hypertension-related morbidity and mortality.^[15]^ African American individuals, for example, have a higher prevalence of hypertension compared to other racial/ethnic groups in the USA, along with higher rates of hypertension-related complications such as stroke, end-stage renal disease (ESRD), and heart failure.^[16]^ Genetic predisposition and social determinants of health, including socioeconomic status, access to healthcare, and environmental factors, contribute to these disparities.^[17]^ Similar disparities in hypertension prevalence and outcomes are observed among other racial and ethnic minority groups globally, highlighting the importance of addressing structural and systemic inequities in healthcare delivery and access. Modifiable lifestyle factors play a crucial role in the development and management of hypertension, with dietary habits, physical activity levels, alcohol consumption, and smoking behavior influencing blood pressure regulation.^[18]^ Diets high in sodium and low in potassium, such as the Western diet, are associated with an increased risk of hypertension, while dietary patterns rich in fruits, vegetables, whole grains, and lean proteins, such as the DASH diet, have been shown to lower blood pressure.^[19]^ Sedentary behavior and insufficient physical activity are independent risk factors for hypertension, with regular exercise exerting beneficial effects on blood pressure through improvements in vascular function, endothelial health, and SNS activity.^[20]^ Excessive alcohol intake and tobacco use are also associated with elevated blood pressure levels and an increased risk of hypertension-related complications, highlighting the importance of lifestyle modification interventions in hypertension prevention and management.

### 4.2. Etiology and pathophysiology

Genetic factors play a significant role in the etiology of hypertension, with evidence suggesting a strong hereditary component in blood pressure regulation.^[2]^ A family history of hypertension is a well-established risk factor for the development of the condition, with individuals having a first-degree relative with hypertension at increased risk compared to those without a family history.^[3]^ Genome-wide association studies have identified numerous genetic variants associated with blood pressure regulation, highlighting hypertension’s polygenic nature and multiple genetic loci involvement.^[4]^ Variations in genes encoding components of the RAAS, SNS, endothelial function, and sodium transport pathways have been implicated in the pathogenesis of hypertension.^[5]^ Environmental factors, including dietary habits, physical inactivity, obesity, and salt intake, contribute significantly to the development of hypertension.^[6]^ Diets high in sodium and low in potassium, such as the Western diet, have been linked to elevated blood pressure levels and an increased risk of hypertension.^[7]^ Excessive sodium intake disrupts the balance of fluid and electrolytes in the body, leading to intravascular volume expansion and arterial vasoconstriction.^[8]^ Conversely, potassium-rich diets benefit blood pressure regulation by promoting natriuresis, vasodilation, and endothelial function.^[9]^ The impact of dietary patterns on hypertension risk underscores the importance of dietary interventions in hypertension prevention and management. Physical inactivity and sedentary behavior are independent risk factors for hypertension, with regular exercise exerting protective effects on blood pressure levels and cardiovascular health.^[10]^ Aerobic exercise, resistance training, and flexibility exercises have been shown to lower blood pressure through various mechanisms, including improvements in vascular function, endothelial nitric oxide bioavailability, and SNS activity.^[11]^ Physical activity also contributes to weight management, insulin sensitivity, and stress reduction, enhancing its beneficial effects on blood pressure regulation.^[12]^ Therefore, regular exercise is recommended as an essential component of hypertension prevention and management strategies. Obesity, particularly central adiposity, and visceral fat accumulation is strongly associated with hypertension and cardiovascular risk.^[13]^ Adipose tissue serves as an endocrine organ, secreting adipokines, and inflammatory cytokines that contribute to insulin resistance, endothelial dysfunction, and dyslipidemia, all promoting hypertension.^[14]^ Additionally, obesity is closely linked to other metabolic abnormalities, including dyslipidemia, hyperglycemia, and insulin resistance, collectively referred to as metabolic syndrome, which further exacerbates cardiovascular risk.^[15]^ Lifestyle modifications targeting weight reduction through caloric restriction, dietary changes, and increased physical activity are effective strategies for lowering blood pressure and reducing cardiovascular risk in obese individuals.^[16]^ Salt sensitivity, characterized by an exaggerated blood pressure response to changes in dietary salt intake, is a common feature of hypertension and may contribute to individual variability in blood pressure regulation.^[17]^ Salt-sensitive individuals exhibit impaired sodium excretion mechanisms, leading to sodium retention, extracellular volume expansion, and increased vascular resistance.^[18]^ Genetic factors, including variations in genes encoding sodium transporters and renal sodium handling mechanisms, play a role in salt sensitivity and hypertension susceptibility.^[19]^ Dietary sodium restriction has been shown to lower blood pressure in salt-sensitive individuals, highlighting the importance of personalized dietary interventions based on individual salt sensitivity status.^[20]^ Chronic stress and psychosocial factors have been implicated in the pathogenesis of hypertension, with chronic activation of the SNS and hypothalamic-pituitary-adrenal axis contributing to blood pressure dysregulation.^[21]^ Psychological stressors, such as job strain, social isolation, financial difficulties, and emotional distress, can lead to increased sympathetic activity, endothelial dysfunction, and inflammation, all of which promote hypertension development and progression.^[22]^ Stress management techniques, including mindfulness meditation, relaxation therapies, and cognitive-behavioral interventions, have been shown to lower blood pressure and improve cardiovascular outcomes by mitigating the effects of chronic stress on the body.^[23]^ The pathophysiology of hypertension encompasses dysregulation of vascular tone, sodium and fluid balance, neurohormonal signaling, endothelial function, and renal hemodynamics, contributing to sustained elevation in systemic arterial pressure.^[2]^ At the vascular level, hypertension is associated with abnormalities in vascular smooth muscle tone, endothelial function, and vascular remodeling, leading to increased peripheral resistance and impaired vasodilation.^[3]^ Dysfunctional endothelium, characterized by reduced nitric oxide bioavailability, enhanced vasoconstrictor tone, and increased oxidative stress, contributes to endothelial dysfunction and impaired vasodilatory capacity in hypertensive individuals.^[4]^ Additionally, vascular remodeling processes, including hypertrophy, fibrosis, and arterial stiffness, further contribute to increased vascular resistance and elevated blood pressure levels.^[5]^ Renal dysfunction plays a central role in the pathophysiology of hypertension, with alterations in renal sodium handling, fluid balance, and renal hemodynamics contributing to blood pressure dysregulation.^[6]^ The RAAS, a key regulator of blood pressure and fluid-electrolyte balance, is often dysregulated in hypertensive individuals, leading to excessive sodium retention, intravascular volume expansion, and vasoconstriction.^[7]^ Angiotensin II, a potent vasoconstrictor peptide, promotes renal sodium reabsorption, aldosterone secretion, and SNS activation, contributing to increased systemic vascular resistance and elevated blood pressure.^[8]^ SNS overactivity is a hallmark feature of hypertension, characterized by increased sympathetic tone, enhanced catecholamine release, and augmented adrenergic receptor responsiveness.^[9]^ Chronic sympathetic activation leads to vasoconstriction, sodium retention, cardiac remodeling, and baroreceptor dysfunction, all contributing to sustained elevation in blood pressure levels.^[10]^ Additionally, central nervous system mechanisms, including alterations in autonomic control centers, neurohormonal signaling pathways, and baroreflex sensitivity, further modulate sympathetic outflow and blood pressure regulation in hypertensive individuals.^[11]^ Dysregulation of sodium and fluid balance is a key pathophysiological mechanism underlying hypertension, with abnormalities in renal sodium handling, extracellular volume expansion, and osmotic pressure contributing to blood pressure dysregulation.^[12]^ Due to enhanced tubular reabsorption or reduced glomerular filtration rate, impaired renal sodium excretion results in sodium retention and intravascular volume expansion, leading to increased cardiac output and elevated blood pressure levels.^[13]^ Additionally, alterations in extracellular fluid volume, osmotic pressure, and tissue perfusion further contribute to blood pressure dysregulation in hypertensive individuals.^[14]^ Endocrine abnormalities, including insulin resistance, dyslipidemia, and abnormalities in adipokine secretion, are commonly observed in individuals with hypertension and contribute to cardiovascular risk.^[15]^ Insulin resistance, characterized by impaired insulin signaling and glucose metabolism, is associated with endothelial dysfunction, sympathetic activation, and vascular inflammation, all promoting hypertension development and progression.^[16]^ Dyslipidemia, characterized by elevated levels of triglycerides, low-density lipoprotein (LDL) cholesterol, and decreased high-density lipoprotein (HDL) cholesterol, contributes to atherosclerosis, endothelial dysfunction, and arterial stiffness, further exacerbating cardiovascular risk in hypertensive individuals.^[17]^ Adipokines, including adiponectin, leptin, and resistin, play a role in modulating vascular function, insulin sensitivity, and inflammatory processes, with dysregulated adipokine signaling contributing to hypertension pathophysiology.^[18]^ Inflammatory and oxidative stress pathways are implicated in the pathophysiology of hypertension, with immune activation, cytokine release, and oxidative damage contributing to endothelial dysfunction, vascular remodeling, and blood pressure dysregulation.^[19]^ Chronic low-grade inflammation, characterized by elevated levels of proinflammatory cytokines, including interleukin-6 (IL-6), tumor necrosis factor-alpha (TNF-α), and C-reactive protein (CRP), promotes vascular dysfunction, insulin resistance, and atherosclerosis, all of which contribute to hypertension development and progression.^[20]^ Oxidative stress, resulting from an imbalance between reactive oxygen species production and antioxidant defense mechanisms, leads to endothelial dysfunction, vascular inflammation, and smooth muscle cell proliferation, further exacerbating hypertension-related complications.^[21]^

### 4.3. Classification of hypertension

The classification of hypertension is essential for diagnosis, risk stratification, and treatment selection, providing clinicians with a framework for understanding the severity and complexity of the condition.^[1]^ Hypertension is typically classified based on blood pressure measurements, presence of comorbidities, and assessment of target organ damage, allowing for tailored management approaches and risk reduction strategies.^[2]^ The most widely used classification system for hypertension is based on blood pressure levels measured in millimeters of mercury (mm Hg). It is defined by guidelines from professional organizations such as the AHA, the ESC, and the National Institute for Health and Care Excellence.^[3]^ According to these guidelines, hypertension is classified into several categories based on SBP and DBP measurements obtained during clinical evaluation.^[4]^ Normal blood pressure is defined as SBP < 120 mm Hg and DBP < 80 mm Hg, indicating optimal cardiovascular health and low risk of hypertension-related complications.^[5]^ Elevated blood pressure, a newly recognized category introduced in recent guidelines, is characterized by SBP ranging from 120 to 129 mm Hg and DBP < 80 mm Hg, representing an increased risk of developing hypertension and cardiovascular disease (CVD) if left untreated.^[6]^ Elevated blood pressure serves as a warning sign for early intervention and lifestyle modifications to prevent progression to hypertension and mitigate cardiovascular risk.^[7]^ Stage 1 hypertension is defined by SBP ranging from 130 to 139 mm Hg or DBP ranging from 80 to 89 mm Hg, indicating mild to moderate elevation in blood pressure levels.^[8]^ Stage 2 hypertension, considered more severe, is characterized by SBP ≥ 140 mm Hg or DBP ≥ 90 mm Hg, indicating significant elevation in blood pressure and increased risk of hypertension-related complications.^[9]^ Hypertension crisis, a life-threatening condition requiring immediate medical attention, is defined by SBP ≥ 180 mm Hg and DBP ≥ 120 mm Hg, accompanied by evidence of acute target organ damage such as hypertensive encephalopathy, acute myocardial infarction, or acute kidney injury.^[10]^ Hypertension crisis requires prompt management with antihypertensive medications and intensive monitoring to prevent further complications and organ dysfunction.^[11]^ In addition to blood pressure measurements, the classification of hypertension considers the presence of comorbidities and assessment of target organ damage, providing additional insights into the severity and complexity of the condition.^[12]^ Hypertension is often classified as primary (essential) or secondary based on the underlying etiology and presence of identifiable causes.^[13]^ Primary hypertension, accounting for the majority of cases, is characterized by elevated blood pressure levels without an identifiable cause, typically attributed to a combination of genetic, environmental, and lifestyle factors.^[14]^ Secondary hypertension, less common but potentially reversible, is characterized by elevated blood pressure levels secondary to underlying medical conditions such as renal artery stenosis (RAS), primary aldosteronism, pheochromocytoma, or Cushing syndrome.^[15]^ Identifying and addressing underlying secondary causes of hypertension is essential for optimizing management and achieving blood pressure control in affected individuals.^[16]^ The classification of hypertension also considers the presence of target organ damage, reflecting the impact of elevated blood pressure on end-organ function and integrity.^[17]^ Target organ damage may manifest in various organ systems, including the heart, brain, kidneys, and vasculature, and is associated with increased cardiovascular risk and mortality.^[18]^ Common manifestations of target organ damage include left ventricular hypertrophy (LVH), ischemic heart disease, heart failure, stroke, transient ischemic attack (TIA), CKD, proteinuria, retinopathy, and peripheral artery disease (PAD).^[19]^ Assessment of target organ damage provides valuable prognostic information and guides treatment decisions, helping to prevent cardiovascular events and improve long-term outcomes in hypertensive individuals.^[20]^

### 4.4. Types of hypertension

(1) Primary hypertension, also known as essential hypertension, represents the predominant form of high blood pressure encountered in clinical practice, constituting approximately 90% to 95% of all diagnosed cases.^[1]^ Unlike secondary hypertension, which can be attributed to identifiable causes such as RAS or primary aldosteronism, primary hypertension arises without a specific underlying cause, making it a diagnosis of exclusion.^[2]^ The pathogenesis of primary hypertension is multifactorial, involving complex interactions between genetic, environmental, and lifestyle factors, as well as alterations in vascular and neurohormonal pathways. Genetic factors play a significant role in the development of primary hypertension, with heritability estimates ranging from 30% to 50%.^[3]^ Numerous genetic variants have been implicated in blood pressure regulation, including polymorphisms in genes encoding for components of the RAAS, adrenergic receptors, sodium channels, and endothelial nitric oxide synthase.^[4]^ Genome-wide association studies have identified multiple single nucleotide polymorphisms associated with blood pressure traits, providing insights into the genetic basis of hypertension susceptibility and potential targets for pharmacogenomic interventions.^[5]^ Environmental and lifestyle factors play a crucial role in the pathogenesis of primary hypertension, contributing to the rising prevalence of the condition worldwide.^[6]^ Dietary factors, such as high sodium intake, low potassium intake, excessive alcohol consumption, and a diet rich in processed foods and saturated fats, are strongly associated with elevated blood pressure levels.^[7]^ A sedentary lifestyle, physical inactivity, obesity, and central adiposity are also significant risk factors for hypertension development, contributing to insulin resistance, dyslipidemia, and endothelial dysfunction.^[8]^ Chronic psychosocial stress, socioeconomic disparities, urbanization, and environmental pollutants further exacerbate the risk of primary hypertension, highlighting the importance of addressing social determinants of health in hypertension prevention and management.^[9]^ Alterations in vascular structure and function contribute to the pathogenesis of primary hypertension, leading to increased peripheral vascular resistance and impaired vasodilatory capacity.^[10]^ Endothelial dysfunction, characterized by reduced nitric oxide bioavailability, enhanced vasoconstrictor tone, and increased oxidative stress, plays a central role in hypertension pathophysiology, promoting vascular inflammation, smooth muscle cell proliferation, and arterial stiffness.^[11]^ Vascular remodeling processes, including hypertrophy, fibrosis, and arteriolar narrowing, further contribute to elevated systemic vascular resistance and blood pressure levels in individuals with primary hypertension.^[12]^ Neurohormonal dysregulation is a hallmark feature of primary hypertension, involving alterations in SNS activity, RAAS activation, and endothelin-1 signaling.^[13]^ Chronic sympathetic overactivity leads to vasoconstriction, sodium retention, and increased heart rate, contributing to elevated blood pressure levels and target organ damage.^[14]^ Dysregulated RAAS activation, characterized by increased renin secretion, angiotensin II production, and aldosterone release, further promotes sodium retention, intravascular volume expansion, and vascular remodeling, exacerbating hypertension-related complications.^[15]^ Endothelin-1, a potent vasoconstrictor peptide, is upregulated in hypertensive individuals, promoting smooth muscle cell proliferation, fibrosis, and vascular inflammation, further contributing to blood pressure dysregulation.^[16]^ The natural history of primary hypertension is often characterized by a prolonged asymptomatic phase, during which blood pressure gradually increases over time without overt clinical manifestations.^[17]^ However, sustained elevation in blood pressure levels predisposes individuals to target organ damage, including LVH, ischemic heart disease, heart failure, stroke, TIA, CKD, proteinuria, retinopathy, and PAD.^[18]^ Complications of primary hypertension arise from the chronic hemodynamic stress imposed on the cardiovascular system, as well as the harmful effects of hypertension on vascular integrity, endothelial function, and organ perfusion. Management of primary hypertension focuses on comprehensive risk assessment, lifestyle modifications, and pharmacological interventions aimed at reducing blood pressure levels and mitigating cardiovascular risk.^[19]^ Lifestyle modifications, including dietary sodium restriction, weight loss, increased physical activity, moderation of alcohol consumption, and adoption of the DASH diet, are recommended as first-line therapy for individuals with primary hypertension.^[20]^ Pharmacological interventions, such as diuretics, ACE inhibitors, ARBs, CCBs, beta-blockers, and mineralocorticoid receptor antagonists, are indicated for individuals with persistent hypertension or those at high cardiovascular risk.^[21]^
(2 )Secondary hypertension, representing approximately 5% to 10% of all diagnosed cases of high blood pressure, differs from primary hypertension in that it arises from identifiable underlying causes or contributing factors.^[1]^ Unlike primary hypertension, which develops gradually over time without a specific etiology, secondary hypertension often has a clear pathophysiological basis. It may be reversible with appropriate treatment of the underlying condition.^[2]^ Secondary hypertension can result from a wide range of medical conditions affecting the renal, endocrine, cardiovascular, and neurological systems, as well as from certain medications or lifestyle factors. RAS represents one of the most common causes of secondary hypertension, particularly in older adults with atherosclerotic vascular disease.^[3]^ RAS occurs when one or both renal arteries become narrowed or obstructed, leading to reduced renal perfusion, RAAS activation, and subsequent elevation in blood pressure levels.^[4]^ Atherosclerosis is the primary underlying mechanism, although other etiologies such as fibromuscular dysplasia, vasculitis, and external compression may also contribute to renal artery narrowing.^[5]^ Diagnosis of RAS typically involves noninvasive imaging modalities such as renal duplex ultrasonography, computed tomography (CT) angiography, magnetic resonance angiography, or renal scintigraphy.^[6]^ Primary aldosteronism, also known as Conn syndrome, represents another common cause of secondary hypertension, accounting for approximately 5% to 10% of hypertensive cases.^[7]^ Primary aldosteronism is characterized by excessive aldosterone production from the adrenal glands, leading to sodium retention, potassium excretion, and volume expansion, resulting in hypertension and hypokalemia.^[8]^ The most common underlying etiology is aldosterone-producing adenoma or unilateral adrenal hyperplasia, although bilateral adrenal hyperplasia may also occur.^[9]^ Diagnosis of primary aldosteronism involves screening tests such as the aldosterone-to-renin ratio followed by confirmatory testing with saline infusion test, oral sodium loading test, or captopril challenge test, along with adrenal imaging to localize aldosterone-producing lesions.^[10]^ Cushing syndrome, caused by excessive cortisol production, represents another endocrine disorder associated with secondary hypertension.^[11]^ Cushing syndrome may result from adrenal adenomas, adrenal carcinomas, or pituitary adenomas secreting adrenocorticotropic hormone (ACTH), as well as ectopic ACTH-secreting tumors.^[12]^ Excess cortisol leads to metabolic abnormalities, including insulin resistance, glucose intolerance, dyslipidemia, central adiposity, and hypertension, contributing to increased cardiovascular risk and target organ damage.^[13]^ Diagnosis of Cushing syndrome involves screening tests such as 24-hour urinary free cortisol measurement, followed by confirmatory testing with low-dose dexamethasone suppression test, late-night salivary cortisol measurement, or ACTH stimulation test, along with imaging studies to localize adrenal or pituitary lesions.^[14]^ Pheochromocytoma and paraganglioma represent rare neuroendocrine tumors arising from chromaffin cells in the adrenal medulla or extra-adrenal paraganglia, respectively, secreting catecholamines such as epinephrine and norepinephrine.^[15]^ Excessive catecholamine release leads to paroxysmal hypertension, palpitations, headache, diaphoresis, and other sympathetic symptoms, as well as increased cardiovascular risk and hypertensive crises.^[16]^ Diagnosis of pheochromocytoma and paraganglioma involves biochemical testing such as plasma or urinary metanephrines and catecholamines, followed by anatomical imaging with CT, magnetic resonance imaging (MRI), or metaiodobenzylguanidine scintigraphy for tumor localization.^[17]^ Obstructive sleep apnea (OSA) represents a common and underdiagnosed medical condition associated with secondary hypertension.^[18]^ OSA is characterized by recurrent episodes of partial or complete upper airway collapse during sleep, leading to intermittent hypoxia, hypercapnia, sympathetic activation, and arousal from sleep, resulting in systemic inflammation, endothelial dysfunction, and metabolic dysregulation.^[19]^ Chronic intermittent hypoxia and sympathetic overactivity contribute to increased blood pressure variability, nocturnal hypertension, and non-dipping blood pressure patterns, exacerbating cardiovascular risk in individuals with OSA.^[20]^ Diagnosis of OSA involves overnight polysomnography or home sleep apnea testing, followed by treatment with continuous positive airway pressure therapy or oral appliances to improve nocturnal respiratory function and reduce blood pressure levels.^[21]^ Certain medications and substances may also contribute to secondary hypertension through various mechanisms, including vasoconstriction, sodium retention, volume expansion, or direct cardiotoxic effects.^[22]^ Common culprits include nonsteroidal anti-inflammatory drugs, oral contraceptives, corticosteroids, sympathomimetic agents, decongestants, erythropoietin, cyclosporine, tacrolimus, and herbal supplements such as licorice and ephedra.^[23]^ Medication-induced hypertension often resolves with discontinuation of the offending agent, although alternative treatments or dose adjustments may be necessary to manage underlying medical conditions.
(3) White-coat hypertension, also known as isolated clinic hypertension or white-coat syndrome, describes a phenomenon where individuals exhibit elevated blood pressure readings in a clinical or medical setting, such as a doctor’s office or clinic, but have normal blood pressure levels in other settings, such as home or ambulatory monitoring.^[3]^ This condition challenges the traditional understanding of hypertension as a consistent and persistent elevation in blood pressure. It raises questions about the accuracy of clinic-based blood pressure measurements in diagnosing and managing hypertension. First described in the early 1980s, white-coat hypertension initially puzzled clinicians and researchers who encountered patients with markedly elevated blood pressure readings in medical settings but normal blood pressure measurements outside of these environments.^[1]^ The term “white coat” refers to the white coats traditionally worn by healthcare providers, symbolizing the association between elevated blood pressure and the clinical environment. White-coat hypertension gained recognition as a distinct clinical entity with the advent of ambulatory blood pressure monitoring (ABPM), which allows for continuous monitoring of blood pressure throughout the day and night in the patient’s natural environment, providing a more comprehensive assessment of blood pressure variability and patterns.^[7]^ The prevalence of white-coat hypertension varies widely depending on the population studied and the diagnostic criteria used. Studies have reported prevalence rates ranging from 10% to 40% among individuals diagnosed with hypertension based on clinic-based blood pressure measurements.^[1]^ White-coat hypertension is more commonly observed in younger individuals, women, nonsmokers, and individuals with lower cardiovascular risk profiles. However, it can occur across all age groups and demographic categories.^[2]^ The diagnosis of white-coat hypertension relies on the demonstration of elevated clinic blood pressure readings (SBP ≥ 140 mm Hg and DBP ≥ 90 mm Hg) on multiple occasions, along with normal blood pressure levels (<135/85 mm Hg) on ambulatory or home blood pressure monitoring (HBPM).^[3]^ The pathophysiology of white-coat hypertension remains incompletely understood but is thought to involve complex interactions between psychological, physiological, and environmental factors. The clinical environment, characterized by unfamiliar surroundings, white-coat-induced anxiety, and the presence of healthcare providers, may trigger the “white-coat effect,” leading to transient increases in blood pressure levels through SNS activation and stress-induced hormonal responses.^[4]^ Psychological factors such as anxiety, apprehension, fear of medical procedures, and previous negative experiences with healthcare providers may further exacerbate the white-coat effect, contributing to blood pressure elevation in susceptible individuals.^[5]^ Physiological mechanisms underlying white-coat hypertension include heightened SNS activity, increased catecholamine release, peripheral vasoconstriction, and alterations in vascular tone and endothelial function.^[6]^ Sympathetic activation leads to enhanced cardiac output, increased heart rate, and peripheral vasoconstriction, resulting in elevated blood pressure levels in response to acute stress or anxiety.^[7]^ Psychological stressors such as the anticipation of medical procedures or interactions with healthcare providers may trigger the release of catecholamines such as epinephrine and norepinephrine, further contributing to blood pressure variability and the white-coat effect.^[8]^ Endothelial dysfunction, characterized by impaired nitric oxide bioavailability and enhanced vasoconstrictor tone, may also play a role in the pathophysiology of white-coat hypertension, contributing to increased peripheral vascular resistance and blood pressure variability.^[9]^ The clinical significance of white-coat hypertension remains a subject of debate, with conflicting evidence regarding its association with cardiovascular outcomes and long-term prognosis. While individuals with white-coat hypertension generally have a lower risk of cardiovascular events compared to those with sustained hypertension, they may still be at increased risk of target organ damage, including LVH, arterial stiffness, and microvascular dysfunction, particularly if blood pressure elevation persists over time or is associated with other cardiovascular risk factors.^[10]^ Studies have reported conflicting findings regarding the impact of white-coat hypertension on cardiovascular morbidity and mortality, with some studies suggesting an increased risk of cardiovascular events and others finding no significant difference compared to normotensive individuals.^[11]^ The presence of masked hypertension, where individuals have normal clinic blood pressure readings but elevated out-of-office blood pressure levels, further complicates the interpretation of cardiovascular risk in individuals with white-coat hypertension.^[12]^ Management of white-coat hypertension involves accurate diagnosis, risk stratification, and individualized treatment strategies based on the patient’s clinical characteristics and cardiovascular risk profile. Lifestyle modifications, including dietary sodium restriction, weight loss, increased physical activity, moderation of alcohol consumption, and stress reduction techniques, are recommended as first-line therapy for individuals with white-coat hypertension, regardless of their cardiovascular risk status.^[13]^ Pharmacological interventions may be considered in select cases, particularly if lifestyle modifications fail to achieve blood pressure control or if individuals have comorbid conditions such as diabetes, CKD, or established CVD.^[14]^ Antihypertensive medications, including diuretics, beta-blockers, CCBs, ACE inhibitors, ARBs, and mineralocorticoid receptor antagonists, may be used cautiously in individuals with white-coat hypertension to prevent target organ damage and reduce cardiovascular risk.^[15]^
(4) Masked hypertension, reverse or occult hypertension, is characterized by normal blood pressure readings in a clinical or medical setting but elevated blood pressure levels outside of this environment, such as during ambulatory or HBPM.^[16]^ This phenomenon challenges the conventional understanding of hypertension as a consistent and persistent elevation in blood pressure and underscores the importance of comprehensive blood pressure assessment beyond clinic-based measurements.^[17]^ First described in the early 1980s, masked hypertension initially puzzled clinicians and researchers who encountered individuals with seemingly normal blood pressure readings in medical settings but elevated blood pressure levels outside of these environments. The term “masked” refers to the hidden nature of hypertension in these individuals, as their true blood pressure status may be obscured by the white-coat effect or other environmental factors present during clinic visits.^[18]^ Masked hypertension gained recognition as a distinct clinical entity with the advent of ABPM, which allows for continuous monitoring of blood pressure throughout the day and night in the patient’s natural environment, providing a more comprehensive assessment of blood pressure variability and patterns.^[2]^ The prevalence of masked hypertension varies widely depending on the population studied, the diagnostic criteria used, and the methodology employed for blood pressure measurement. Studies have reported prevalence rates ranging from 8% to 30% among individuals with normal clinic blood pressure readings, with higher rates observed in specific subgroups such as older adults, men, individuals with obesity, diabetes, or CKD, and those with a family history of hypertension or CVD.^[19]^ The diagnosis of masked hypertension relies on the demonstration of normal clinic blood pressure readings (SBP < 140 mm Hg and DBP < 90 mm Hg) on multiple occasions, along with elevated blood pressure levels (≥135/85 mm Hg) on ambulatory or HBPM. The pathophysiology of masked hypertension remains incompletely understood but is thought to involve similar mechanisms as white-coat hypertension, including psychological, physiological, and environmental factors.^[20]^ Individuals with masked hypertension may exhibit normal blood pressure readings in a medical setting due to the absence of stressors or triggers that typically provoke the white-coat effect, such as anxiety, apprehension, or fear of medical procedures. However, in their natural environment, away from the clinical setting, these individuals may experience chronic stress, sedentary behavior, poor dietary habits, and other lifestyle factors contributing to elevated blood pressure levels over time.^[21]^ Physiological mechanisms underlying masked hypertension may include SNS activation, endothelial dysfunction, and vascular tone and compliance alterations. Chronic stress and psychological factors such as job strain, marital conflict, financial worries, or social isolation may trigger SNS activation, leading to increased catecholamine release, peripheral vasoconstriction, and blood pressure elevation.^[22]^ Endothelial dysfunction, characterized by impaired nitric oxide bioavailability and enhanced vasoconstrictor tone, may also play a role in the pathophysiology of masked hypertension, contributing to increased peripheral vascular resistance and blood pressure variability.^[23]^ The clinical significance of masked hypertension remains a subject of debate, with conflicting evidence regarding its association with cardiovascular outcomes and long-term prognosis. While individuals with masked hypertension generally have a lower risk of cardiovascular events compared to those with sustained hypertension, they may still be at increased risk of target organ damage, including LVH, arterial stiffness, and microvascular dysfunction, particularly if blood pressure elevation persists over time or is associated with other cardiovascular risk factors.^[24]^ Studies have reported conflicting findings regarding the impact of masked hypertension on cardiovascular morbidity and mortality, with some studies suggesting an increased risk of cardiovascular events and others finding no significant difference compared to normotensive individuals. The diagnosis and management of masked hypertension require accurate identification of individuals at risk and implementation of targeted interventions to reduce cardiovascular risk.^[25]^ Risk stratification tools such as the masked hypertension risk score may help identify individuals at increased risk of masked hypertension and guide decision-making regarding ambulatory or HBPM. Lifestyle modifications, including dietary sodium restriction, weight loss, increased physical activity, stress reduction techniques, and smoking cessation, are recommended as first-line therapy for individuals with masked hypertension, regardless of their cardiovascular risk status.^[26]^ Pharmacological interventions may be considered in select cases, particularly if lifestyle modifications fail to achieve blood pressure control or if individuals have comorbid conditions such as diabetes, CKD, or established CVD.
(5) Resistant hypertension is a challenging clinical condition characterized by persistently elevated blood pressure levels despite adherence to optimal lifestyle modifications and treatment with appropriate antihypertensive medications.^[3]^ The condition poses significant diagnostic and therapeutic dilemmas for healthcare providers and affects a substantial proportion of individuals with hypertension worldwide.^[27]^ Resistant hypertension is associated with increased cardiovascular morbidity and mortality, including higher rates of stroke, myocardial infarction, heart failure, CKD, and premature death. Understanding the underlying mechanisms, risk factors, diagnostic evaluation, and management strategies for resistant hypertension is essential for optimizing patient outcomes and reducing the burden of CVD.^[28]^ The prevalence of resistant hypertension varies depending on the population studied, the diagnostic criteria used, and the methodology employed for blood pressure measurement. Studies have reported prevalence rates ranging from 10% to 20% among individuals with treated hypertension, with higher rates observed in specific subgroups such as older adults, women, individuals with obesity, diabetes, CKD, or OSA, and those with a history of CVD or target organ damage.^[1]^ Resistant hypertension is more common in individuals with uncontrolled blood pressure levels, suboptimal adherence to antihypertensive medications, inadequate treatment intensity, or comorbid conditions contributing to hypertension pathogenesis. The pathophysiology of resistant hypertension is multifactorial and may involve complex interactions between genetic, environmental, lifestyle, and clinical factors.^[29]^ Potential mechanisms underlying resistant hypertension include excessive sodium intake, volume overload, SNS activation, RAAS overactivity, endothelial dysfunction, insulin resistance, obesity, OSA, CKD, and medication non-adherence or resistance. Genetic factors may also contribute to variability in blood pressure response to antihypertensive medications, influencing treatment outcomes and therapeutic efficacy.^[30]^ Diagnostic evaluation of resistant hypertension requires a comprehensive assessment of potential contributing factors, including medication history, adherence to lifestyle modifications and pharmacological therapy, identification of secondary causes or comorbid conditions, assessment of target organ damage, evaluation of medication adherence and resistance, and ambulatory or HBPM.^[31]^ ABPM is recommended to confirm the diagnosis of resistant hypertension and assess blood pressure variability, nocturnal dipping patterns, and white-coat or masked hypertension. Laboratory tests such as serum electrolytes, renal function, lipid profile, fasting glucose, and urine albumin-to-creatinine ratio may be indicated to evaluate for secondary causes or comorbid conditions contributing to hypertension pathogenesis.^[3]^ Management of resistant hypertension involves a multidisciplinary approach incorporating lifestyle modifications, pharmacological interventions, and targeted therapies aimed at achieving blood pressure control and reducing cardiovascular risk. Lifestyle modifications, including dietary sodium restriction, weight loss, increased physical activity, moderation of alcohol consumption, smoking cessation, stress reduction techniques, and adherence to the DASH diet, are recommended as first-line therapy for individuals with resistant hypertension, regardless of their cardiovascular risk status.^[4]^ Pharmacological interventions for resistant hypertension may include optimization of antihypertensive medications, intensification of treatment regimens, use of combination therapies, addition of complementary agents targeting specific mechanisms of hypertension pathogenesis, and consideration of device-based therapies or interventional procedures.^[19]^ Antihypertensive medications commonly used in the management of resistant hypertension include diuretics, CCBs, ACE inhibitors, ARBs, beta-blockers, aldosterone receptor antagonists, direct renin inhibitors, centrally acting agents, alpha-blockers, vasodilators, and sympatholytic agents. Treatment selection should be individualized based on patient characteristics, comorbidities, medication tolerability, adverse effects, cost considerations, and evidence-based guidelines.^[10]^

### 4.5. Effects on health

CVD represents the most common and severe complication of hypertension, encompassing a spectrum of conditions, including coronary artery disease, heart failure, arrhythmias, and peripheral vascular disease (PVD).^[3]^ Hypertension promotes atherosclerosis, endothelial dysfunction, and arterial stiffness, predisposing individuals to myocardial infarction, angina pectoris, and sudden cardiac death.^[4]^ Chronic pressure overload on the heart leads to LVH, diastolic dysfunction, and impaired contractility, increasing the risk of heart failure and cardiovascular mortality.^[5]^ Additionally, hypertension is a major risk factor for atrial fibrillation, ventricular arrhythmias, and other cardiac arrhythmias, further contributing to cardiovascular morbidity and mortality.^[6]^ Stroke, both ischemic and hemorrhagic, represents a devastating consequence of hypertension, accounting for a significant proportion of cerebrovascular events worldwide.^[7]^ Chronic hypertension promotes cerebrovascular remodeling, endothelial dysfunction, and small vessel disease, increasing the risk of thrombotic and embolic events in cerebral circulation.^[8]^ Hypertension-related intracerebral hemorrhage results from the rupture of fragile vessels due to chronic hypertension-induced vascular remodeling and alterations in cerebral autoregulation, leading to significant neurological deficits and mortality.^[9]^

Additionally, hypertension is a significant risk factor for TIAs, cognitive impairment, and vascular dementia, further exacerbating the burden of cerebrovascular disease.^[10]^ Renal dysfunction is a common complication of hypertension, with chronic uncontrolled hypertension leading to progressive renal damage, proteinuria, and declining renal function.^[11]^ Hypertensive nephropathy, characterized by glomerulosclerosis, tubulointerstitial fibrosis, and renal arteriolar narrowing, is a leading cause of ESRD worldwide.^[12]^ Elevated blood pressure levels promote renal microvascular injury, glomerular hypertension, and podocyte dysfunction, leading to proteinuria, glomerular sclerosis, and progressive loss of renal function.^[13]^

Additionally, hypertension exacerbates the progression of diabetic nephropathy, CKD, and other renal disorders, further increasing the risk of ESRD and cardiovascular complications.^[14]^ PVD represents another significant health consequence of hypertension, characterized by reduced blood flow to the extremities, impaired wound healing, and increased risk of limb amputation.^[15]^ Chronic hypertension promotes atherosclerosis, endothelial dysfunction, and arterial stiffness in peripheral arteries, leading to claudication, ischemic ulcers, and gangrene.^[16]^ Additionally, hypertension is associated with an increased risk of abdominal aortic aneurysms, carotid artery disease, and other peripheral vascular disorders, further contributing to morbidity and mortality.^[17]^ Hypertension exerts systemic effects beyond the cardiovascular system, influencing metabolic, inflammatory, and neuroendocrine pathways, leading to adverse health outcomes.^[18]^ Insulin resistance, dyslipidemia, and obesity are commonly observed in individuals with hypertension, contributing to the development of type 2 diabetes mellitus, metabolic syndrome, and increased cardiovascular risk.^[19]^ Chronic inflammation, characterized by elevated levels of proinflammatory cytokines, including IL-6, TNF-α, and CRP, promotes endothelial dysfunction, insulin resistance, and atherosclerosis, further exacerbating cardiovascular risk in hypertensive individuals.^[20]^ Dysregulated neuroendocrine pathways, including the RAAS and SNS, contribute to systemic vasoconstriction, sodium retention, and oxidative stress, further exacerbating hypertension-related complications.^[21]^ In addition to its direct effects on physical health, hypertension also exerts significant psychosocial and quality-of-life impacts, contributing to stress, anxiety, depression, and impaired social functioning.^[22]^ Individuals with hypertension often experience reduced quality of life, impaired work productivity, and increased healthcare utilization, leading to a substantial economic burden on healthcare systems and society.^[23]^ Furthermore, hypertension-related complications, including stroke, heart failure, and renal dysfunction, impose significant caregiving responsibilities on family members and caregivers, further exacerbating the psychosocial and economic impact of the disease.^[24]^

## 5. Diagnosis of hypertension

Medical history plays a central role in diagnosing hypertension, as it provides essential information about the patient’s past medical conditions, family history, lifestyle habits, medication use, and symptoms suggestive of secondary causes or comorbidities.^[1]^ Key elements of the medical history include age, gender, race/ethnicity, smoking status, alcohol consumption, dietary habits, physical activity level, stress levels, medication adherence, and presence of symptoms such as headache, dizziness, chest pain, palpitations, shortness of breath, or visual disturbances.^[2]^ A family history of hypertension, CVD, CKD, diabetes, or other hereditary conditions may also influence the likelihood of developing hypertension and guide diagnostic evaluation.^[3]^ Physical examination is another integral component of diagnosing hypertension, as it allows healthcare providers to assess vital signs, cardiovascular status, and signs of target organ damage. Key elements of the physical examination include measurement of blood pressure, assessment of body mass index, waist circumference, and signs of obesity or central adiposity, palpation of peripheral pulses and signs of arterial stiffness, auscultation of heart and lung sounds for murmurs or abnormal breath sounds, inspection of fundoscopic findings for signs of retinopathy, and assessment of peripheral edema or signs of heart failure.^[4]^ Abnormal findings on physical examination may suggest underlying secondary causes or comorbid conditions contributing to hypertension pathogenesis and guide further diagnostic evaluation. Blood pressure measurement is the cornerstone of diagnosing hypertension and involves accurate assessment of SBP and DBP using standardized techniques and calibrated equipment.^[5]^ Blood pressure should be measured in a quiet, temperature-controlled environment, with the patient seated, feet flat on the floor, arm supported at heart level, and back supported against a chair. The appropriate cuff size should be selected based on the patient’s arm circumference, with the bladder encircling at least 80% of the arm circumference. Multiple blood pressure readings should be obtained at each visit, with at least 2 readings taken 1 minute apart, and the average of these readings should be used to determine blood pressure levels.^[6]^ Home blood pressure or ABPM may be recommended for individuals with suspected white-coat hypertension, masked hypertension, or resistant hypertension to assess blood pressure variability and patterns. Assessment of cardiovascular risk factors is essential for identifying individuals at increased risk of developing hypertension and guiding preventive strategies.^[7]^ Common cardiovascular risk factors include age, gender, race/ethnicity, family history of hypertension or CVD, smoking status, obesity, physical inactivity, unhealthy diet, excessive alcohol consumption, dyslipidemia, diabetes, CKD, OSA, and psychosocial stress. The presence of multiple cardiovascular risk factors or comorbid conditions may indicate a higher likelihood of hypertension and warrant more aggressive risk factor modification and treatment interventions.^[8]^ Evaluation of target organ damage is critical for assessing the impact of hypertension on end-organ function and guiding therapeutic decision-making.^[9]^ Target organ damage may manifest as LVH, arterial stiffness, atherosclerosis, coronary artery disease, stroke, TIA, heart failure, CKD, proteinuria, microalbuminuria, retinopathy, PAD, cognitive impairment, or dementia.^[10]^ Diagnostic tests such as electrocardiography (ECG), echocardiography, carotid ultrasound, coronary artery calcium scoring, brain imaging (e.g., MRI, CT), renal ultrasound, urine analysis, and ophthalmoscopy may be indicated to assess for signs of target organ damage and inform prognosis and management.^[11]^ Imaging studies such as echocardiography, ECG, and carotid ultrasound play a vital role in assessing target organ damage and cardiovascular risk in individuals with hypertension. Echocardiography allows for noninvasive cardiac structure and function evaluation, providing information about LVH, diastolic dysfunction, systolic dysfunction, valvular abnormalities, and pericardial disease.^[12]^ ECG assesses electrical conduction and rhythm disturbances, identifying arrhythmias, conduction abnormalities, and signs of myocardial ischemia or infarction. Carotid ultrasound evaluates carotid intima-media thickness and identifies carotid plaque, providing insights into subclinical atherosclerosis and cardiovascular risk. Advanced imaging modalities such as CT, MRI, and nuclear imaging techniques may be indicated in select cases to assess for secondary causes of hypertension, identify target organ damage, and guide therapeutic interventions.^[13]^ CT angiography and magnetic resonance angiography allow for noninvasive evaluation of the renal arteries, identifying RAS or other vascular abnormalities contributing to hypertension pathogenesis.^[14]^ Renal ultrasound assesses renal size, cortical thickness, and parenchymal echogenicity, identifying structural abnormalities such as renal cysts, tumors, or hydronephrosis. Nuclear imaging techniques such as renography, captopril renography, and scintigraphy may assess renal perfusion, function, and responsiveness to pharmacological interventions in individuals with suspected renovascular hypertension.^[15]^ Specialized procedures such as ABPM, HBPM, and provocative testing may be employed to assess blood pressure variability, nocturnal dipping patterns, and response to physiological or pharmacological stimuli.^[16]^ ABPM allows continuous blood pressure monitoring throughout the day and night, providing insights into blood pressure variability, white-coat hypertension, masked hypertension, and circadian rhythm disturbances. HBPM involves self-measurement of blood pressure at home using automated devices, complementing clinic-based measurements and providing a more comprehensive assessment of blood pressure control.^[17]^ Provocative testing, such as the captopril challenge test or saline infusion test, may be used to assess for secondary causes of hypertension, including aldosterone-producing adenoma, renovascular hypertension, or pheochromocytoma.^[18]^

## 6. Lifestyle modifications

Dietary changes, particularly adherence to the DASH diet, are pivotal in managing hypertension and reducing cardiovascular risk.^[19]^ The DASH diet is a dietary pattern rich in fruits, vegetables, whole grains, lean proteins, and low-fat dairy products, focusing on reducing sodium intake and limiting saturated and trans fats. Numerous studies have demonstrated the effectiveness of the DASH diet in lowering blood pressure levels, improving lipid profiles, and reducing the risk of cardiovascular events. The DASH diet emphasizes the consumption of nutrient-dense foods rich in potassium, magnesium, calcium, fiber, and antioxidants, which have been shown to have beneficial effects on blood pressure regulation and cardiovascular health.^[1]^ Fruits and vegetables, particularly those high in potassium, such as bananas, oranges, potatoes, spinach, and tomatoes, help lower blood pressure by promoting diuresis, vasodilation, and sodium excretion through their high potassium and low sodium content. Whole grains, such as brown rice, quinoa, oats, and whole wheat bread, provide dietary fiber and magnesium, which help regulate blood pressure by improving insulin sensitivity, reducing oxidative stress, and enhancing endothelial function.^[5]^ Lean proteins, including poultry, fish, beans, legumes, and nuts, are preferred protein sources on the DASH diet, as they are lower in saturated fats and cholesterol than red meats and processed meats. Plant-based proteins such as beans, lentils, chickpeas, and tofu are also rich in fiber, phytochemicals, and antioxidants, which have cardioprotective effects and may help lower blood pressure levels. Low-fat dairy products such as skim milk, yogurt, and cheese are included in moderation on the DASH diet, providing essential nutrients such as calcium, vitamin D, and potassium without contributing excessive saturated fats or calories.^[10]^ Sodium reduction is a cornerstone of the DASH diet, as high sodium intake is strongly associated with hypertension and increased cardiovascular risk. The DASH-Sodium trial demonstrated that reducing dietary sodium intake to 1500 mg per day, in conjunction with the DASH dietary pattern, resulted in greater reductions in blood pressure compared to either intervention alone. Sodium restriction is achieved by minimizing the use of salt during cooking and at the table, avoiding processed and packaged foods high in sodium, and choosing fresh, whole foods prepared with herbs, spices, and other flavorings instead of salt.^[3]^ The DASH diet also emphasizes the importance of portion control, moderation, and balanced eating patterns to achieve and maintain healthy body weight and blood pressure levels. Portion sizes are controlled to ensure appropriate calorie intake and prevent excessive energy consumption, which can contribute to weight gain and hypertension.^[8]^ Balanced eating patterns involve consuming various foods from all food groups in appropriate proportions, emphasizing nutrient-rich foods that provide essential vitamins, minerals, and antioxidants for optimal health. Numerous clinical trials and observational studies have demonstrated the beneficial effects of the DASH diet on blood pressure reduction and cardiovascular risk reduction.^[9]^ The original DASH trial showed that adherence to the dietary pattern significantly lowered blood pressure levels in individuals with hypertension, with greater reductions observed in those with higher baseline blood pressure levels. Subsequent studies have confirmed the effectiveness of the DASH diet in lowering blood pressure levels in diverse populations, including adults, children, older adults, individuals with diabetes, and those at increased cardiovascular risk.^[5]^ In addition to its effects on blood pressure regulation, the DASH diet has been shown to improve other cardiovascular risk factors, including lipid profiles, insulin sensitivity, inflammation, oxidative stress, and endothelial function.^[1]^ The DASH-Sodium trial demonstrated that combining the DASH dietary pattern with sodium reduction resulted in greater improvements in lipid profiles, particularly LDL cholesterol and triglycerides, compared to either intervention alone. Other studies have shown that the DASH diet is associated with a reduced risk of developing type 2 diabetes, stroke, heart failure, and other cardiovascular events. Regular exercise is a cornerstone of hypertension management and cardiovascular health, offering numerous benefits for blood pressure regulation, endothelial function, vascular health, and overall well-being. Physical activity has been shown to lower blood pressure levels, improve lipid profiles, enhance insulin sensitivity, reduce inflammation, promote weight loss, and reduce the risk of cardiovascular events.^[3]^ Incorporating regular exercise into daily routines is essential for individuals with hypertension to achieve and maintain optimal blood pressure control and reduce the risk of complications associated with uncontrolled hypertension. Aerobic exercise, also known as cardiorespiratory or endurance exercise, involves rhythmic and continuous movements that increase heart rate and breathing rate, such as walking, jogging, cycling, swimming, dancing, and aerobic classes. Aerobic exercise has lowered blood pressure levels by improving cardiac output, reducing peripheral vascular resistance, enhancing endothelial function, and promoting vasodilation.^[8]^ Regular aerobic exercise increases stroke volume, cardiac output, and oxygen delivery to tissues, improving cardiovascular efficiency and reducing the workload on the heart. Aerobic exercise also stimulates the production of nitric oxide, a potent vasodilator, which helps relax blood vessels and improve blood flow, reducing blood pressure levels and enhancing vascular health. Resistance exercise, also known as strength training or weightlifting, involves using resistance or weights to build muscle strength, endurance, and power. Resistance exercise has been shown to lower blood pressure levels by increasing muscle mass, improving insulin sensitivity, reducing visceral adiposity, and enhancing metabolic rate.^[4]^ Regular resistance exercise increases muscle protein synthesis, muscle fiber recruitment, and muscle mass, which contributes to improved glucose metabolism, lipid profiles, and blood pressure regulation. Resistance exercise also increases nitric oxide production and blood flow to working muscles, promoting vasodilation and reducing vascular resistance, which lowers blood pressure levels and improves cardiovascular health.^[4]^ Flexibility exercise, also known as stretching or range-of-motion exercises, involves gentle and controlled movements that improve joint mobility, flexibility, and muscle elasticity.^[5]^ Flexibility exercise can lower blood pressure levels by reducing muscle tension, improving circulation, and promoting relaxation. Regular flexibility exercise increases joint range of motion, muscle flexibility, and tissue elasticity, which reduces the risk of musculoskeletal injuries and enhances overall physical function. Flexibility exercise also reduces stress, tension, and anxiety, common triggers for hypertension and cardiovascular events, promoting mental and emotional well-being.^[6]^ The American College of Sports Medicine and the AHA recommend that adults engage in moderate-intensity aerobic exercise for at least 150 minutes per week or vigorous-intensity aerobic exercise for at least 75 minutes per week, along with muscle-strengthening activities on ≥2 days per week.^[1]^ Moderate-intensity aerobic exercise is equivalent to brisk walking, while vigorous-intensity aerobic exercise is equivalent to jogging or running. Muscle-strengthening activities include resistance exercises using body weight, free weights, resistance bands, or weight machines.^[7]^ Regular flexibility exercises should be performed to improve joint range of motion and prevent musculoskeletal injuries. The benefits of regular exercise extend beyond blood pressure reduction to include improvements in lipid profiles, glucose metabolism, body composition, mental health, and quality of life. Regular physical activity has been shown to lower LDL cholesterol levels, increase HDL cholesterol levels, and reduce triglyceride levels, which lowers the risk of atherosclerosis, coronary artery disease, and stroke.^[8]^ Exercise also improves insulin sensitivity, glucose uptake, and glycemic control, reducing the risk of type 2 diabetes and metabolic syndrome. Regular exercise promotes weight loss, fat loss, and muscle gain, which improves body composition, metabolic rate, and physical function. Exercise has also been shown to have positive effects on mental health, reducing symptoms of depression, anxiety, stress, and cognitive decline. Regular physical activity releases endorphins, serotonin, and dopamine, neurotransmitters that improve mood, reduce pain perception, and promote relaxation.^[9]^ Exercise also enhances cognitive, memory, and executive function, which may help reduce the risk of dementia and cognitive decline with aging. Regular exercise improves quality of life, enhances well-being, and promotes healthy aging. Stress management techniques play a crucial role in the management of hypertension and overall cardiovascular health, as chronic stress is a significant risk factor for the development and exacerbation of hypertension, CVD, and other chronic conditions.^[5]^ Stress activates the SNS, triggering the release of catecholamines and cortisol, which increase heart rate, blood pressure, and vascular tone, promoting vasoconstriction and hypertension.^[10]^ Chronic stress also leads to maladaptive coping behaviors such as overeating, smoking, alcohol consumption, sedentary lifestyle, poor sleep habits, and medication non-adherence, which further exacerbate hypertension and cardiovascular risk. Implementing effective stress management techniques can help individuals with hypertension reduce stress levels, improve coping skills, enhance resilience, and promote overall well-being.^[11]^ One of the most widely studied stress management techniques is relaxation therapy, which involves various relaxation techniques such as deep breathing, progressive muscle relaxation, guided imagery, mindfulness meditation, and autogenic training. These techniques promote relaxation responses in the body, activating the parasympathetic nervous system and counteracting the effects of stress on the cardiovascular system. Deep breathing exercises involve slow, deep, and rhythmic breathing patterns, which increase oxygenation, decrease heart rate, and induce a sense of calm and relaxation.^[12]^ Progressive muscle relaxation involves systematically tensing and relaxing different muscle groups, reducing muscle tension, and promoting physical and mental relaxation. Guided imagery uses visualization and mental imagery to evoke pleasant and calming sensations, promoting relaxation and stress reduction. Mindfulness meditation involves focusing on the moment without judgment, accepting thoughts and feelings as they arise, and cultivating awareness and equanimity in daily life.^[13]^ Autogenic training uses self-suggestions and affirmations to induce relaxation responses in the body, promoting feelings of warmth, heaviness, and relaxation. Cognitive-behavioral therapy (CBT) is another effective stress management technique that helps individuals identify and challenge maladaptive thought patterns, beliefs, and behaviors contributing to stress and anxiety.^[14]^ CBT techniques include cognitive restructuring, problem-solving, assertiveness training, relaxation training, and stress inoculation training, which help individuals develop coping skills, improve self-efficacy, and build resilience in the face of stressors. Cognitive restructuring involves identifying and challenging negative or irrational thoughts and replacing them with more adaptive and realistic interpretations.^[15]^ Problem-solving techniques involve breaking down stressful situations into manageable steps, generating alternative solutions, and implementing effective coping strategies to address challenges. Assertiveness training helps individuals communicate their needs, preferences, and boundaries assertively and effectively, reducing interpersonal conflict and stress. Relaxation training teaches individuals relaxation techniques such as deep breathing, progressive muscle relaxation, and visualization to promote physical and mental relaxation in response to stressors.^[16]^ Stress inoculation training involves exposing individuals to mild stressors in a controlled setting, teaching them adaptive coping skills and strategies to manage stress effectively and build resilience over time. Physical activity and exercise are effective stress management techniques that help reduce stress levels, improve mood, and promote overall well-being. Exercise activates the release of endorphins, serotonin, and dopamine, neurotransmitters that improve mood, reduce pain perception, and promote relaxation.^[17]^ Regular physical activity has been shown to lower cortisol levels, decrease SNS activity, and increase parasympathetic nervous system activity, which reduces stress and anxiety levels. Exercise also distracts from stressors, enhances self-esteem and self-efficacy, and promotes social interaction and support, which buffer against the negative effects of stress on mental and emotional health. Walking, jogging, cycling, swimming, dancing, yoga, tai chi, and qigong reduce stress and promote relaxation.^[18]^ Social support and interpersonal relationships play a critical role in stress management and coping with hypertension and CVD. Strong social support networks provide emotional support, practical assistance, and encouragement during stress, helping individuals cope more effectively and adaptively to stressors. Spending time with friends, family, and loved ones, engaging in enjoyable activities, and participating in social gatherings and community events can help reduce stress levels, improve mood, and enhance overall well-being.^[19]^ Support groups, counseling, and therapy can also provide valuable resources and assistance for individuals coping with hypertension and CVD, helping them develop coping skills, improve communication, and build resilience in the face of stressors. Limiting alcohol and caffeine intake is an important aspect of managing hypertension and promoting cardiovascular health. Both alcohol and caffeine consumption have been associated with increases in blood pressure and cardiovascular risk, and reducing their intake can help individuals with hypertension achieve better blood pressure control and reduce the risk of complications.^[20]^ Understanding the effects of alcohol and caffeine on blood pressure regulation and cardiovascular health, as well as implementing strategies to limit their consumption, is essential for optimizing hypertension management and overall well-being. Alcohol consumption has complex effects on blood pressure regulation, with both acute and chronic intake influencing blood pressure levels.^[21]^ Acute alcohol consumption can lead to a transient increase in blood pressure, attributed to its vasodilatory and diuretic effects, as well as activation of the SNS.^[22]^ Chronic alcohol consumption, on the other hand, has been associated with sustained elevations in blood pressure, as well as an increased risk of hypertension, CVD, stroke, heart failure, and arrhythmias. The mechanisms underlying the hypertensive effects of chronic alcohol consumption include increased SNS activity, activation of the RAAS, endothelial dysfunction, oxidative stress, inflammation, and direct toxic effects on the cardiovascular system.^[23]^ Heavy alcohol consumption, defined as >2 drinks per day for men and >1 drink per day for women, has been shown to increase the risk of hypertension and cardiovascular events significantly. In contrast, moderate alcohol consumption, defined as up to 1 drink per day for women and up to 2 drinks per day for men, may have neutral or modestly beneficial effects on cardiovascular health.^[24]^ Caffeine is a central nervous system stimulant found in coffee, tea, energy drinks, soft drinks, chocolate, and certain medications. Caffeine consumption has acute effects on blood pressure, with transient increases observed shortly after ingestion, attributed to its vasoconstrictive and sympathomimetic effects.^[25]^ Caffeine acts as a competitive antagonist of adenosine receptors, blocking the inhibitory effects of adenosine on SNS activity, leading to increased catecholamine release and vasoconstriction.^[26]^ Chronic caffeine consumption has been associated with tolerance development, as well as alterations in vascular tone, endothelial function, and cardiovascular risk. Regular caffeine consumption may lead to sustained elevations in blood pressure, particularly in individuals with hypertension or CVD. It may increase the risk of adverse cardiovascular events, including myocardial infarction, stroke, and arrhythmias.^[27]^ The hypertensive effects of caffeine are dose-dependent, with higher doses associated with greater increases in blood pressure, and individual susceptibility to caffeine varies based on genetic factors, age, sex, race/ethnicity, comorbid conditions, and medication use. Limiting alcohol and caffeine intake is recommended as part of a comprehensive approach to managing hypertension and reducing cardiovascular risk.^[28]^ Strategies for limiting alcohol consumption include setting limits on alcohol intake, avoiding binge drinking, spacing out drinks, alternating alcoholic and nonalcoholic beverages, diluting drinks with water or mixer, avoiding high-alcohol content beverages, and avoiding situations that may trigger excessive drinking. For individuals with hypertension or CVD, it is advisable to limit alcohol consumption to moderate levels or abstain from alcohol altogether to minimize the risk of adverse cardiovascular events.^[29]^ Strategies for limiting caffeine consumption include reducing the number of caffeinated beverages consumed per day, choosing decaffeinated or low-caffeine alternatives, avoiding caffeine-containing products later in the day, limiting caffeine intake during pregnancy or breastfeeding, and monitoring caffeine consumption from all sources, including coffee, tea, energy drinks, soft drinks, chocolate, and medications.^[30]^ For individuals with hypertension or CVD, it is advisable to limit caffeine consumption to moderate levels or avoid caffeine altogether, particularly if caffeine exacerbates symptoms or increases blood pressure levels.^[31]^ Smoking cessation is a critical component of hypertension management and cardiovascular health, as smoking is a major modifiable risk factor for the development and progression of hypertension, CVD, and other chronic conditions.^[6]^ Cigarette smoking is associated with numerous adverse effects on the cardiovascular system, including endothelial dysfunction, oxidative stress, inflammation, atherosclerosis, arterial stiffness, thrombosis, and vasoconstriction, which contribute to the pathogenesis of hypertension and increase the risk of cardiovascular events.^[9]^ Quitting smoking has immediate and long-term benefits for blood pressure control, cardiovascular health, and overall well-being, making smoking cessation a top priority for individuals with hypertension and those at risk of CVD. Cigarette smoking is a well-established risk factor for hypertension, with smoking increasing the risk of developing hypertension by approximately 50% compared to nonsmokers.^[10]^ Smoking is associated with acute increases in blood pressure due to the nicotine content of cigarettes, which stimulates the release of catecholamines, such as epinephrine and norepinephrine, leading to SNS activation, vasoconstriction, and increased heart rate.^[11]^ Chronic smoking is associated with sustained elevations in blood pressure, as well as adverse changes in vascular structure and function, including endothelial dysfunction, arterial stiffness, and atherosclerosis. Smoking cessation has been shown to lead to reductions in blood pressure levels, improvements in endothelial function, and reversal of vascular damage, reducing the risk of hypertension and cardiovascular events over time.^[12]^ The benefits of smoking cessation extend beyond blood pressure reduction to include improvements in cardiovascular risk factors, including lipid profiles, insulin sensitivity, inflammation, and oxidative stress.^[13]^ Quitting smoking leads to improvements in lipid profiles, with increases in HDL cholesterol levels and reductions in LDL cholesterol levels, triglycerides, and total cholesterol levels, which lower the risk of atherosclerosis and coronary artery disease. Smoking cessation also improves insulin sensitivity and glucose metabolism, reducing the risk of insulin resistance, type 2 diabetes, and metabolic syndrome.^[14]^ Quitting smoking reduces inflammation and oxidative stress, with decreases in circulating inflammatory markers such as CRP, IL-6, and TNF-α, which lower the risk of atherosclerosis, plaque rupture, and thrombosis.^[14]^ Quitting smoking has immediate and long-term benefits for cardiovascular health, with reductions in the risk of myocardial infarction, stroke, heart failure, PAD, and sudden cardiac death observed within months of smoking cessation. The benefits of smoking cessation are dose-dependent, with greater reductions in cardiovascular risk observed in individuals who quit smoking completely compared to those who continue to smoke or reduce their smoking intensity.^[15]^ The benefits of smoking cessation are also cumulative, with each year of abstinence leading to further reductions in cardiovascular risk and improvements in overall health and quality of life. Counseling, behavioral therapy, pharmacotherapy, and support services are effective interventions for smoking cessation, helping individuals quit smoking successfully and maintain abstinence over the long term.^[16]^ Counseling and behavioral therapy focus on identifying triggers for smoking, developing coping skills, building motivation to quit, setting goals for smoking cessation, and implementing strategies to overcome barriers to quitting. Behavioral interventions include CBT, motivational interviewing, problem-solving therapy, social support, and stress management techniques, which help individuals develop healthy coping mechanisms, improve self-efficacy, and build resilience in the face of nicotine cravings and withdrawal symptoms.^[17]^ Pharmacotherapy includes nicotine replacement therapy, such as nicotine patches, gum, lozenges, inhalers, and nasal sprays, which deliver controlled doses of nicotine to reduce withdrawal symptoms and cravings, as well as prescription medications such as bupropion (Zyban) and varenicline (Chantix), which help reduce nicotine dependence and withdrawal symptoms by acting on neurotransmitter systems in the brain.^[18]^ Support services such as quitlines, online forums, support groups, and community programs provide additional resources and assistance for individuals quitting smoking, offering counseling, education, referrals, and peer support to help individuals navigate the challenges of smoking cessation and maintain abstinence over the long term. Multidisciplinary collaboration, patient education, shared decision-making, and regular follow-up are essential components of comprehensive care for individuals quitting smoking, helping them overcome barriers to quitting, manage withdrawal symptoms, prevent relapse, and achieve long-term success in maintaining smoking abstinence.^[19,20]^

## 7. Pharmacological interventions

### 7.1. Classification of antihypertensives

Diuretics are among the oldest and most commonly used classes of antihypertensive drugs. They increase sodium and water excretion by the kidneys, reducing extracellular fluid volume and blood pressure.^[1]^ Thiazide diuretics, such as hydrochlorothiazide and chlorthalidone, are commonly used as first-line agents in the treatment of hypertension, particularly in individuals with uncomplicated hypertension or volume overload.^[2]^ Loop diuretics, such as furosemide and bumetanide, are reserved for individuals with more severe hypertension or renal dysfunction. Potassium-sparing diuretics, such as spironolactone and eplerenone, are often combined with thiazide or loop diuretics to prevent hypokalemia and minimize electrolyte disturbances.^[3]^ Beta-blockers are another class of antihypertensive drugs that work by blocking the effects of catecholamines, such as epinephrine and norepinephrine, on beta-adrenergic receptors in the heart and blood vessels.^[4]^ Beta-blockers decrease heart rate, myocardial contractility, and cardiac output by reducing SNS activity and blood pressure. Beta-blockers also dilate blood vessels and decrease renin release, contributing to their antihypertensive effects.^[5]^ Commonly used beta-blockers include atenolol, metoprolol, propranolol, and carvedilol, each with varying selectivity for beta-adrenergic receptors and additional effects on other receptor subtypes.^[6]^ CCBs are a diverse class of antihypertensive drugs that inhibit the influx of calcium ions into vascular smooth muscle cells, leading to vasodilation and reduced peripheral vascular resistance.^[7]^ CCBs are classified into 2 main subclasses based on their mechanisms of action: dihydropyridines and non-dihydropyridines. Dihydropyridine CCBs, such as amlodipine, nifedipine, and felodipine, primarily act on L-type calcium channels in vascular smooth muscle cells, leading to vasodilation and reduced blood pressure.^[8]^ Non-dihydropyridine CCBs, such as verapamil and diltiazem, also inhibit calcium channels in the heart, leading to negative chronotropic and dromotropic effects and vasodilation. CCBs are effective antihypertensive agents, particularly in individuals with isolated systolic hypertension or coronary artery disease.^[9]^ ACE inhibitors are a class of antihypertensive drugs that inhibit the conversion of angiotensin I to angiotensin II, a potent vasoconstrictor, by blocking the action of ACE. ACE inhibitors promote vasodilation, decrease aldosterone secretion, and increase sodium and water excretion by reducing blood pressure and angiotensin II levels. Commonly used ACE inhibitors include lisinopril, enalapril, ramipril, and captopril, each with similar efficacy and side effect profiles.^[10]^ ACE inhibitors are particularly effective in individuals with hypertension and concomitant heart failure, diabetes, or CKD, as they provide renoprotective and cardioprotective effects beyond blood pressure reduction.^[11]^ ARBs are another class of antihypertensive drugs that block the effects of angiotensin II on angiotensin type 1 receptors, leading to vasodilation and reduced blood pressure. ARBs are structurally similar to ACE inhibitors but act on a different target in the RAAS.^[12]^ Commonly used ARBs include losartan, valsartan, irbesartan, and candesartan, each with similar efficacy and tolerability. ARBs are often used as alternatives to ACE inhibitors in individuals who are intolerant of ACE inhibitors due to cough or angioedema, as well as in individuals with hypertension and concomitant heart failure, diabetes, or CKD.^[13]^ Direct renin inhibitors are a newer class of antihypertensive drugs that block the activity of renin, the rate-limiting enzyme in the RAAS. By inhibiting renin, these medications reduce the production of angiotensin I and II, leading to vasodilation and reduced blood pressure. Aliskiren is the only direct renin inhibitor currently available for the treatment of hypertension, either as monotherapy or in combination with other antihypertensive agents.^[14]^ Direct renin inhibitors are generally well-tolerated and effective in lowering blood pressure, particularly in individuals with hypertension and concomitant cardiovascular risk factors. Alpha-adrenergic blockers are a class of antihypertensive drugs that block the effects of catecholamines on alpha-adrenergic receptors in peripheral blood vessels, leading to vasodilation and reduced blood pressure.^[15]^ Alpha-adrenergic blockers are particularly effective in reducing peripheral vascular resistance and improving symptoms of benign prostatic hyperplasia in men. Commonly used alpha-adrenergic blockers include doxazosin, prazosin, and terazosin, each with similar efficacy and side effect profiles. Alpha-adrenergic blockers are often used as adjunctive therapy in individuals with hypertension who do not achieve adequate blood pressure control with other antihypertensive agents.^[16]^ Central alpha agonists are antihypertensive drugs that act on central nervous system receptors to reduce SNS activity, leading to vasodilation and reduced blood pressure. Central alpha agonists decrease sympathetic outflow from the brainstem, leading to decreased peripheral vascular resistance, heart rate, and cardiac output.^[17]^ Commonly used central alpha agonists include clonidine, methyldopa, and guanfacine, each with varying selectivity for central alpha receptors and additional effects on other neurotransmitter systems. Central alpha agonists are effective antihypertensive agents, particularly in individuals with hypertension and concomitant sympathetic overactivity, such as those with resistant hypertension or autonomic dysfunction.^[18]^ Direct vasodilators are antihypertensive drugs that act directly on vascular smooth muscle cells to promote vasodilation and reduce blood pressure. Direct vasodilators relax vascular smooth muscle cells by activating potassium channels, inhibiting calcium influx, or enhancing nitric oxide release, decreasing peripheral vascular resistance, and increasing blood flow.^[18]^ Hydralazine and minoxidil are the 2 main direct vasodilators used to treat hypertension, each with unique mechanisms of action and side effect profiles. Direct vasodilators are often used as adjunctive therapy in individuals with severe hypertension or hypertensive emergencies, particularly in those with heart failure or renal dysfunction.^[19]^ Combination therapy involves using ≥2 antihypertensive agents with complementary mechanisms of action to achieve optimal blood pressure control. Combination therapy allows for lower doses of each medication, reducing the risk of side effects and improving tolerability. Commonly used combinations include thiazide diuretics with ACE inhibitors, ARBs, CCBs, and beta-blockers with diuretics or ACE inhibitors.^[20]^ Fixed-dose combination products are available for many antihypertensive agents, simplifying treatment regimens and improving adherence. Combination therapy is particularly effective in individuals with hypertension and multiple cardiovascular risk factors or comorbid conditions, as well as in those who do not achieve adequate blood pressure control with monotherapy.^[21]^ Table 1 overviews various antihypertensive medications and their respective classes, including examples of drugs, mechanisms of action, efficacy, side effects, and patient adherence rates.

**Table 1:** Summary of antihypertensive medications and classes.

Medication class	Examples of drugs	Mechanism of action	Efficacy	Side effects	Patient adherence rates
Diuretics	Hydrochlorothiazide	Increase excretion of sodium and water by the kidneys	Effective in reducing blood volume and peripheral resistance	Electrolyte imbalances (e.g., hypokalemia), dehydration	Moderate to high
	Chlorthalidone			Muscle cramps, weakness, dizziness	
Beta-blockers	Atenolol	Block beta-adrenergic receptors, reducing sympathetic activity	Effective in lowering heart rate and cardiac output	Fatigue, dizziness, bradycardia, sexual dysfunction	Moderate to high
	Metoprolol			Bronchoconstriction, depression, sleep disturbances	
Calcium channel blockers	Amlodipine	Inhibit calcium influx into vascular smooth muscle cells	Effective in reducing peripheral vascular resistance	Peripheral edema, flushing, dizziness	Moderate to high
	Nifedipine			Headache, gingival hyperplasia, reflex tachycardia	
ACE inhibitors	Lisinopril	Inhibit conversion of angiotensin I to angiotensin II	Effective in reducing angiotensin II levels and vasodilation	Dry cough, hyperkalemia, renal dysfunction	Moderate to high
	Enalapril			Angioedema, hypotension, rash	
ARBs	Losartan	Block angiotensin II receptors, leading to vasodilation	Effective in reducing angiotensin II effects	Hyperkalemia, dizziness, renal dysfunction	Moderate to high
	Valsartan			Hypotension, cough, hyperuricemia	
Direct renin inhibitors	Aliskiren	Inhibit renin activity, reducing angiotensin I and II levels	Effective in lowering renin-angiotensin-aldosterone system	Hyperkalemia, diarrhea, angioedema	Moderate to high
Alpha-adrenergic blockers	Doxazosin	Block alpha-adrenergic receptors, leading to vasodilation	Effective in reducing peripheral vascular resistance	Dizziness, orthostatic hypotension, fatigue	Moderate to high
	Prazosin			Headache, nasal congestion, palpitations	
Central alpha agonists	Clonidine	Stimulate central alpha receptors, reducing sympathetic activity	Effective in lowering sympathetic outflow from the brainstem	Dry mouth, sedation, rebound hypertension	Moderate to high
	Methyldopa			Bradycardia, depression, liver dysfunction	
Direct vasodilators	Hydralazine	Activate potassium channels, leading to vasodilation	Effective in reducing peripheral vascular resistance	Headache, lupus-like syndrome, tachycardia	Moderate to high
	Minoxidil			Hypertrichosis, fluid retention, pericardial effusion	
Combination therapy	Hydrochlorothiazide +	Combined effects of individual agents on blood pressure	Effective in achieving blood pressure goals	Side effects of individual medications, potential drug	Moderate to high
	Lisinopril			Interactions, pill burden	

Adherence rates vary depending on factors such as drug regimen complexity, side effects, patient education, and support systems. Generally, adherence rates are moderate to high for most antihypertensive medications, particularly when patients receive comprehensive education, regular follow-up, and support from healthcare providers. However, individual patient factors and preferences should be considered when selecting antihypertensive medications to optimize adherence and treatment outcomes.

ACE = angiotensin-converting enzyme, ARB = angiotensin II receptor blocker.

### 7.2. Potential side effects and considerations

One common class of antihypertensive medications is diuretics, such as thiazide diuretics. While effective in reducing blood pressure by promoting diuresis and decreasing fluid volume, diuretics can lead to electrolyte imbalances, particularly hypokalemia, and hyponatremia.^[22]^ Patients taking diuretics should be monitored regularly for signs of electrolyte disturbances, and potassium-sparing diuretics or supplementation may be considered in some cases. CCBs are another widely used class of antihypertensive medications. They work by inhibiting calcium influx into vascular smooth muscle cells, resulting in vasodilation and decreased blood pressure.^[23]^ However, CCBs can cause side effects such as peripheral edema, flushing, headache, and constipation. Patients experiencing troublesome side effects may benefit from dose adjustments or switching to a different class of medication. ACE inhibitors and ARBs are commonly prescribed for their RAAS inhibiting effects.^[24]^

While these medications are generally well-tolerated, they can cause dry cough, hyperkalemia, and angioedema in some individuals. Patients with a history of angioedema or renal impairment may require closer monitoring when initiating ACE inhibitors or ARBs.^[25]^ Beta-blockers are another antihypertensive medication class that blocks beta-adrenergic receptors, leading to decreased heart rate and cardiac output. Common side effects of beta-blockers include fatigue, dizziness, bradycardia, and worsening symptoms in patients with bronchospastic diseases. Selective beta-blockers may be preferred in patients with asthma or chronic obstructive pulmonary disease to minimize the risk of bronchospasm.^[26]^ Mineralocorticoid receptor antagonists, such as spironolactone, are sometimes used as adjunctive therapy in patients with resistant hypertension or heart failure. These medications can cause hyperkalemia, gynecomastia, and menstrual irregularities. Close monitoring of potassium levels is essential in patients taking mineralocorticoid receptor antagonists, particularly those with renal impairment or concomitant use of other potassium-sparing medications.^[27]^ Adherence to antihypertensive medications is crucial for achieving optimal blood pressure control and reducing the risk of cardiovascular events. However, medication adherence can be challenging for some patients due to factors such as the complexity of treatment regimens, cost of medications, side effects, and lack of understanding about the importance of hypertension management.^[28]^ Healthcare providers should engage in patient education, counseling, and shared decision-making to address barriers to adherence and promote treatment success.^[29]^ In addition to medication-related considerations, lifestyle factors can influence blood pressure control and treatment outcomes. Patients should be encouraged to adopt healthy lifestyle habits, such as following a balanced diet, engaging in regular physical activity, maintaining a healthy weight, limiting alcohol intake, and avoiding tobacco use. These lifestyle modifications can complement pharmacological therapy and help optimize blood pressure control.^[10]^

### 7.3. Guidelines for medication use

Guidelines for medication use in the management of hypertension are essential for optimizing treatment outcomes and reducing the risk of cardiovascular events. Expert panels and professional organizations develop these guidelines based on the best evidence from clinical trials, observational studies, and systematic reviews.^[5]^ They provide recommendations for the selection, initiation, titration, and monitoring of antihypertensive medications and strategies for addressing treatment-resistant hypertension, adherence issues, and special populations.^[6]^ The American College of Cardiology (ACC) and the AHA jointly released updated hypertension guidelines in 2017, marking a significant shift in diagnosing and managing high blood pressure in adults. These guidelines, titled the “2017 ACC/AHA Hypertension Guidelines,” introduced several key changes aimed at improving the detection and treatment of hypertension, ultimately reducing the risk of cardiovascular events and improving patient outcomes.^[1]^ One of the most notable changes introduced by the 2017 guidelines was the redefinition of hypertension. The guidelines lowered the threshold for diagnosing hypertension, defining it as a SBP ≥ 130 mm Hg or a DBP ≥ 80 mm Hg. This lower threshold reflects growing evidence linking even modest elevations in blood pressure to increased cardiovascular risk, particularly among individuals with comorbid conditions such as diabetes, CKD, and established CVD.^[4]^ The guidelines also emphasized the importance of accurate blood pressure measurement techniques, including validated devices, appropriate cuff sizes, and standardized protocols for measurement. ABPM and HBPM were recommended to confirm the diagnosis of hypertension and assess blood pressure variability outside of the clinical setting.^[5]^ These methods provide valuable information about a patient’s blood pressure patterns and help guide treatment decisions. Another key aspect of the 2017 guidelines emphasized lifestyle modifications as first-line therapy for individuals with elevated blood pressure.^[6]^ Lifestyle interventions, including dietary changes (such as the DASH diet), regular exercise, weight management, smoking cessation, and limiting alcohol intake, were recommended for all individuals with hypertension, regardless of whether pharmacological treatment was initiated.^[7]^ For individuals with stage 1 hypertension (SBP 130–139 mm Hg or DBP 80–89 mm Hg) and clinical CVD or 10-year atherosclerotic CVD risk ≥ 10%, initiation of antihypertensive medication was recommended along with lifestyle modifications.^[2]^ The choice of antihypertensive medication was based on individual patient characteristics, including age, race/ethnicity, comorbid conditions, renal function, and cardiovascular risk factors. Thiazide diuretics, CCBs, ACE inhibitors, and ARBs were recommended as first-line agents for most adults, with beta-blockers and aldosterone antagonists considered as alternative options.^[3]^ Combination therapy with ≥2 antihypertensive agents may be required to achieve blood pressure goals, particularly in individuals with stage 2 hypertension (SBP ≥ 140 mm Hg or DBP ≥ 90 mm Hg) or high cardiovascular risk. Regular blood pressure monitoring, adherence to medication regimens, and assessment of treatment response were highlighted as essential components of hypertension management.^[4]^ Regular follow-up visits should be scheduled to assess blood pressure control, evaluate for adverse effects, adjust medication dosages as needed, and reinforce lifestyle modifications. Patient education, shared decision-making, and engagement in self-management strategies were emphasized to promote medication adherence and achieve treatment goals.^[5]^ The 2017 ACC/AHA Hypertension Guidelines provided a comprehensive framework for diagnosing and managing hypertension in adults, emphasizing the importance of accurate blood pressure measurement, lifestyle modifications, and appropriate pharmacological treatment. These guidelines represented a significant step forward in addressing the global burden of hypertension and reducing the risk of CVD worldwide.^[6]^ The ESC and the European Society of Hypertension (ESH) jointly released updated guidelines for managing arterial hypertension in 2018. These guidelines, titled the “2018 ESC/ESH Guidelines for the Management of Arterial Hypertension,” provide evidence-based recommendations for diagnosing, evaluating, and treating hypertension in European populations.^[7]^ The guidelines aim to improve clinical outcomes, reduce cardiovascular morbidity and mortality, and optimize hypertension management strategies across diverse patient populations. One of the key features of the 2018 ESC/ESH guidelines is the emphasis on accurate blood pressure measurement techniques. Like the American guidelines, the ESC/ESH guidelines recommend using validated devices, appropriate cuff sizes, and standardized protocols for blood pressure measurement.^[8]^ ABPM and HBPM are also recommended for confirming the diagnosis of hypertension and assessing blood pressure variability outside of the clinical setting. The guidelines provide updated definitions and classifications of hypertension, including categorizing blood pressure levels and identifying high-risk groups. Hypertension is defined as a SBP ≥ 140 mm Hg or a DBP ≥ 90 mm Hg, based on office measurements.^[9]^ Elevated blood pressure is defined as SBP 130 to 139 mm Hg or DBP 80 to 89 mm Hg, reflecting the increased cardiovascular risk associated with these levels. Like the American guidelines, the ESC/ESH guidelines emphasize lifestyle modifications as first-line therapy for individuals with elevated blood pressure.^[11]^ Lifestyle interventions, including dietary changes (such as the Mediterranean diet), regular physical activity, weight management, smoking cessation, and moderation of alcohol intake, are recommended for all individuals with hypertension, irrespective of whether pharmacological treatment is initiated.^[3]^ For individuals with hypertension requiring pharmacological treatment, the guidelines recommend a stepped-care approach based on individual patient characteristics and cardiovascular risk factors. Thiazide diuretics, CCBs, ACE inhibitors, and ARBs are recommended as first-line agents for most patients, with beta-blockers, mineralocorticoid receptor antagonists, and centrally acting agents considered as alternative options.^[4]^ Individual patient characteristics, including age, race/ethnicity, comorbid conditions, renal function, and cardiovascular risk factors, guide the choice of antihypertensive medication. Combination therapy with ≥2 antihypertensive agents may be necessary to achieve blood pressure goals, particularly in individuals with stage 2 hypertension or high cardiovascular risk.^[5]^ Regular monitoring of blood pressure, adherence to medication regimens, and assessment of treatment response are essential components of hypertension management according to the ESC/ESH guidelines. Regular follow-up visits should be scheduled to assess blood pressure control, evaluate for adverse effects, adjust medication dosages as needed, and reinforce lifestyle modifications.^[6]^ Patient education, shared decision-making, and engagement in self-management strategies are emphasized to promote medication adherence and achieve treatment goals. The 2018 ESC/ESH Guidelines for the Management of Arterial Hypertension represent a comprehensive and evidence-based approach to hypertension management in European populations.^[7]^ By providing updated recommendations for the diagnosis, evaluation, and treatment of hypertension, these guidelines aim to improve clinical outcomes and reduce the burden of CVD across diverse patient populations. The Joint National Committee (JNC) guidelines have significantly shaped the approach to diagnosing and managing hypertension in the USA.^[8]^ Established in 1972, the JNC provided evidence-based recommendations for preventing, detecting, evaluating, and treating high blood pressure. Over the years, the JNC guidelines underwent several revisions to incorporate new evidence and advancements in hypertension research and clinical practice. The most recent version of the JNC guidelines, JNC 8, was released in 2014.^[9]^ These guidelines differed from previous iterations in several key areas, including blood pressure thresholds for diagnosis and treatment, treatment targets, and medication selection. One of the notable changes introduced by JNC 8 was the relaxation of blood pressure targets for certain patient populations.^[20]^ The guidelines recommended a target blood pressure of <140/90 mm Hg for most adults, including those with diabetes or CKD. This represented a shift from previous guidelines, which had recommended lower targets for these high-risk groups. JNC 8 also emphasized the importance of individualizing treatment decisions based on patient characteristics and preferences.^[21]^ The guidelines highlighted the need to consider factors such as age, race/ethnicity, comorbid conditions, and cardiovascular risk when selecting antihypertensive medications and determining treatment goals.^[22]^ In terms of medication selection, JNC 8 recommended a stepped-care approach to treatment, starting with thiazide diuretics, CCBs, ACE inhibitors, or ARBs as first-line agents. Beta-blockers were no longer recommended as first-line therapy, except in certain patient populations such as those with heart failure or postmyocardial infarction.^[23]^ Combination therapy with ≥2 antihypertensive agents was recommended for individuals with stage 2 hypertension (SBP ≥ 140 mm Hg or DBP ≥ 90 mm Hg) or high cardiovascular risk.^[24]^ The choice of antihypertensive medication was guided by individual patient characteristics and included consideration of potential drug interactions, side effects, and cost. JNC 8 also highlighted the importance of lifestyle modifications as a cornerstone of hypertension management.^[3]^ Lifestyle interventions, including dietary changes (such as the DASH diet), regular physical activity, weight management, smoking cessation, and moderation of alcohol intake, were recommended for all individuals with hypertension, irrespective of whether pharmacological treatment was initiated.^[2]^ Regular blood pressure monitoring, adherence to medication regimens, and assessment of treatment response were essential components of hypertension management, according to JNC 8. Regular follow-up visits should be scheduled to assess blood pressure control, evaluate for adverse effects, adjust medication dosages as needed, and reinforce lifestyle modifications.^[5]^ Patient education, shared decision-making, and engagement in self-management strategies were emphasized to promote medication adherence and achieve treatment goals. The WHO guidelines on hypertension are informed by the latest scientific evidence and expert consensus, focusing on promoting equitable access to high-quality healthcare services and interventions for the prevention and control of hypertension.^[6]^ These guidelines address key areas such as the definition and classification of hypertension, strategies for screening and diagnosis, approaches to treatment and management, and recommendations for population-level interventions. One of the fundamental aspects of the WHO guidelines on hypertension is the definition and classification of high blood pressure. Hypertension is defined as a SBP ≥ 140 mm Hg or a DBP ≥ 90 mm Hg, based on office measurements.^[5]^ Elevated blood pressure is defined as SBP 130 to 139 mm Hg or DBP 80 to 89 mm Hg, reflecting the increased cardiovascular risk associated with these levels. The guidelines emphasize the importance of accurate blood pressure measurement techniques, including validated devices, appropriate cuff sizes, and standardized protocols for measurement.^[4]^ ABPM and HBPM are recommended for confirming the diagnosis of hypertension and assessing blood pressure variability outside of the clinical setting. Screening and early detection of hypertension are critical components of the WHO guidelines, as early intervention can prevent or delay the onset of cardiovascular complications.^[5]^ The guidelines recommend population-based screening programs to identify individuals at risk of hypertension and facilitate timely diagnosis and treatment. Screening should be integrated into primary healthcare settings and coupled with lifestyle modification and risk factor management strategies. Regarding treatment and management, the WHO guidelines advocate for a comprehensive approach that addresses pharmacological and non-pharmacological interventions.^[7]^ Lifestyle modifications, including dietary changes (such as reducing sodium intake and adopting a healthy diet), regular physical activity, weight management, smoking cessation, and moderation of alcohol consumption, are recommended as first-line therapy for individuals with elevated blood pressure. The guidelines recommend a stepped-care approach for individuals requiring pharmacological treatment based on patient characteristics, cardiovascular risk factors, and comorbid conditions. Antihypertensive medications, such as thiazide diuretics, CCBs, ACE inhibitors, and ARBs, are recommended as first-line agents for most patients.^[8]^ The choice of antihypertensive medication should be guided by factors such as age, race/ethnicity, comorbid conditions, renal function, and cardiovascular risk profile. Combination therapy with ≥2 antihypertensive agents may be necessary to achieve blood pressure goals, particularly in individuals with stage 2 hypertension or high cardiovascular risk.^[9]^ According to the WHO guidelines, monitoring blood pressure, adherence to medication regimens, and assessment of treatment response are essential components of hypertension management. Regular follow-up visits should be scheduled to assess blood pressure control, evaluate for adverse effects, adjust medication dosages as needed, and reinforce lifestyle modifications.^[10]^ The International Society of Hypertension (ISH) is a global organization dedicated to advancing research, education, and clinical practice in hypertension. The ISH develops guidelines to provide evidence-based recommendations for preventing, diagnosing, and managing high blood pressure, aiming to improve health outcomes and reduce the burden of CVD worldwide.^[11]^ The ISH guidelines on hypertension are developed by expert panels comprised of leading researchers, clinicians, and public health experts. These guidelines integrate the latest scientific evidence, clinical trial data, and expert consensus to provide comprehensive and practical recommendations for healthcare professionals and policymakers. One of the key priorities of the ISH guidelines is to establish clear definitions and classifications of hypertension.^[12]^ Hypertension is defined as a SBP ≥ 140 mm Hg or a DBP ≥ 90 mm Hg, based on office measurements. Elevated blood pressure is defined as SBP 130 to 139 mm Hg or DBP 80 to 89 mm Hg, reflecting the increased cardiovascular risk associated with these levels.^[13]^ Accurate blood pressure measurement techniques are emphasized in the ISH guidelines, including validated devices, appropriate cuff sizes, and standardized protocols for measurement. ABPM and HBPM are recommended for confirming the diagnosis of hypertension and assessing blood pressure variability outside of the clinical setting.^[14]^ The ISH guidelines highlight the importance of early detection and management of hypertension to prevent cardiovascular complications. Screening programs are recommended to identify individuals at risk of hypertension and facilitate timely diagnosis and treatment. Screening should be integrated into primary healthcare settings and targeted at high-risk populations, such as older adults, individuals with comorbid conditions, and those with a family history of hypertension.^[15]^ Regarding treatment and management, the ISH guidelines advocate for a holistic approach that addresses pharmacological and non-pharmacological interventions. Lifestyle modifications, including dietary changes (such as reducing sodium intake and adopting a healthy diet), regular physical activity, weight management, smoking cessation, and moderation of alcohol consumption, are recommended as first-line therapy for individuals with elevated blood pressure.^[16]^ The ISH guidelines recommend a personalized approach for individuals requiring pharmacological treatment based on patient characteristics, cardiovascular risk factors, and comorbid conditions. Antihypertensive medications, such as thiazide diuretics, CCBs, ACE inhibitors, and ARBs, are recommended as first-line agents for most patients.^[17]^ The choice of antihypertensive medication should be guided by factors such as age, race/ethnicity, comorbid conditions, renal function, and cardiovascular risk profile. Combination therapy with ≥2 antihypertensive agents may be necessary to achieve blood pressure goals, particularly in individuals with stage 2 hypertension or high cardiovascular risk.^[18]^ Regular monitoring of blood pressure, adherence to medication regimens, and assessment of treatment response are essential components of hypertension management according to the ISH guidelines. Regular follow-up visits should be scheduled to assess blood pressure control, evaluate for adverse effects, adjust medication dosages as needed, and reinforce lifestyle modifications.^[19]^ The Canadian Hypertension Education Program (CHEP) is a national initiative to improve the prevention, diagnosis, and management of hypertension in Canada. Established in 1999, CHEP provides evidence-based guidelines and educational resources to healthcare professionals, policymakers, and the public to promote best practices in hypertension care. The CHEP guidelines are developed by an expert panel of leading hypertension researchers, clinicians, and public health experts across Canada.^[20]^ These guidelines are updated annually to incorporate the latest scientific evidence, clinical trial data, and expert consensus on hypertension management. One of the primary objectives of the CHEP guidelines is to provide clear definitions and classifications of hypertension. Hypertension is defined as a SBP ≥ 140 mm Hg or a DBP ≥ 90 mm Hg, based on office measurements.^[21]^ Elevated blood pressure is defined as SBP 130 to 139 mm Hg or DBP 80 to 89 mm Hg, reflecting the increased cardiovascular risk associated with these levels. Accurate blood pressure measurement techniques are emphasized in the CHEP guidelines, including validated devices, appropriate cuff sizes, and standardized protocols for measurement.^[22]^ ABPM and HBPM are recommended for confirming the diagnosis of hypertension and assessing blood pressure variability outside of the clinical setting. The CHEP guidelines highlight the importance of early detection and management of hypertension to prevent cardiovascular complications. Screening programs are recommended to identify individuals at risk of hypertension and facilitate timely diagnosis and treatment.^[23]^ Screening should be integrated into primary healthcare settings and targeted at high-risk populations, such as older adults, individuals with comorbid conditions, and those with a family history of hypertension. Regarding treatment and management, the CHEP guidelines advocate for a comprehensive approach that addresses pharmacological and non-pharmacological interventions.^[24]^ Lifestyle modifications, including dietary changes (such as the DASH diet), regular physical activity, weight management, smoking cessation, and moderation of alcohol consumption, are recommended as first-line therapy for individuals with elevated blood pressure. The CHEP guidelines recommend a personalized approach for individuals requiring pharmacological treatment based on patient characteristics, cardiovascular risk factors, and comorbid conditions. Antihypertensive medications, such as thiazide diuretics, CCBs, ACE inhibitors, and ARBs, are recommended as first-line agents for most patients.^[25]^ The choice of antihypertensive medication should be guided by factors such as age, race/ethnicity, comorbid conditions, renal function, and cardiovascular risk profile. Combination therapy with ≥2 antihypertensive agents may be necessary to achieve blood pressure goals, particularly in individuals with stage 2 hypertension or high cardiovascular risk.^[26]^ Regular monitoring of blood pressure, adherence to medication regimens, and assessment of treatment response are essential components of hypertension management according to the CHEP guidelines. Regular follow-up visits should be scheduled to assess blood pressure control, evaluate for adverse effects, adjust medication dosages as needed, and reinforce lifestyle modifications.^[27]^ Table 2 offers a concise overview of key guidelines and recommendations for hypertension management, including information on target blood pressure goals, lifestyle modifications, pharmacological interventions, and treatment strategies recommended by major healthcare organizations and professional societies.

**Table 2:** Summary of hypertension guidelines and recommendations.

Guideline	Blood pressure definition	Diagnosis	Lifestyle modifications	Pharmacological treatment	Monitoring and follow-up
ACC/AHA guidelines	SBP ≥ 130 mm Hg or DBP ≥ 80 mm Hg	Accurate measurement methods	Dietary changes (e.g., DASH diet), regular exercise, weight management	Thiazide diuretics, CCBs, ACE inhibitors, ARBs, beta-blockers, aldosterone antagonists	Regular assessment of BP control, medication adherence
ESC/ESH guidelines	SBP ≥ 140 mm Hg or DBP ≥ 90 mm Hg	Integrated into primary care	Dietary changes (e.g., Mediterranean diet), physical activity	Thiazide diuretics, CCBs, ACE inhibitors, ARBs, beta-blockers, mineralocorticoid receptor antagonists	Regular monitoring of BP, adherence to treatment
JNC guidelines	SBP ≥ 140 mm Hg or DBP ≥ 90 mm Hg	Population-based screening	Lifestyle modifications (e.g., DASH diet), weight management	Thiazide diuretics, CCBs, ACE inhibitors, ARBs, beta-blockers, aldosterone antagonists	Regular follow-up visits for assessment
WHO guidelines	SBP ≥ 140 mm Hg or DBP ≥ 90 mm Hg	Targeted at high-risk groups	Lifestyle modifications (e.g., sodium reduction, healthy diet)	Thiazide diuretics, CCBs, ACE inhibitors, ARBs, beta-blockers	Regular monitoring of BP, adherence to treatment
ISH guidelines	SBP ≥ 140 mm Hg or DBP ≥ 90 mm Hg	Early detection programs	Lifestyle modifications (e.g., dietary changes, physical activity)	Thiazide diuretics, CCBs, ACE inhibitors, ARBs, beta-blockers	Regular assessment of BP control, medication adherence
CHEP guidelines	SBP ≥ 140 mm Hg or DBP ≥ 90 mm Hg	Integrated into primary care	Lifestyle modifications (e.g., DASH diet), smoking cessation	Thiazide diuretics, CCBs, ACE inhibitors, ARBs, beta-blockers	Regular monitoring of BP, adherence to treatment

This table provides a comparative overview of the key recommendations across different hypertension guidelines, including definitions, diagnosis, lifestyle modifications, pharmacological treatment options, and monitoring/follow-up strategies. Each guideline emphasizes the importance of accurate blood pressure measurement, lifestyle modifications, and personalized treatment approaches based on individual patient characteristics and cardiovascular risk factors.

ACC = American College of Cardiology, ACE = angiotensin-converting enzyme, AHA = American Heart Association, ARB = angiotensin II receptor blocker, BP = blood pressure, CCB = calcium channel blocker, CHEP = Canadian Hypertension Education Program, DASH = Dietary Approaches to Stop Hypertension, DBP = diastolic blood pressure, ESC = European Society of Cardiology, ESH = European Society of Hypertension, ISH = International Society of Hypertension, JNC = Joint National Committee, SBP = systolic blood pressure, WHO = World Health Organization,

## 8. Complementary approaches

The use of herbal remedies and supplements for various health conditions, including hypertension, has gained popularity in recent years. Many individuals seek alternative or complementary therapies to conventional medications, often motivated by concerns about side effects, desire for natural treatments, or cultural beliefs.^[28]^ While some herbal remedies and supplements may have potential benefits for managing hypertension, it is essential to approach their use with caution and evidence-based knowledge.^[29]^ Herbal remedies and supplements encompass a wide range of botanical products, including herbs, plants, roots, and extracts, that are purported to have medicinal properties. Some of the most commonly used herbal remedies for hypertension include garlic, hawthorn, hibiscus, green tea, and olive leaf extract.^[30]^ These botanicals have been studied for their potential effects on blood pressure regulation, vascular health, and cardiovascular risk reduction. Garlic (Allium sativum) is one of the most extensively studied herbal remedies for hypertension. It contains bioactive compounds such as allicin, which have been shown to have vasodilatory, antioxidant, and antihypertensive effects.^[31]^ Clinical trials investigating the effects of garlic supplementation on blood pressure have yielded mixed results, with some studies suggesting modest reductions in systolic and DBP compared to placebo. However, the overall evidence for garlic’s efficacy in hypertension management remains inconclusive, and further research is needed to clarify its role. Hawthorn (Crataegus spp.) is another herbal remedy traditionally used for cardiovascular health, including hypertension.^[3]^ Hawthorn extracts are rich in flavonoids and oligomeric proanthocyanidins, which have antioxidant and vasodilatory properties. Some studies have suggested that hawthorn supplementation may modestly lower blood pressure and improve endothelial function in individuals with hypertension.^[4]^ However, the evidence for hawthorn’s efficacy as a standalone treatment for hypertension is limited, and its use should be approached cautiously, especially in individuals taking antihypertensive medications. Hibiscus (Hibiscus sabdariffa) is a flowering plant commonly consumed as a herbal tea in many cultures.^[32]^ Hibiscus tea is rich in polyphenols, including anthocyanins and flavonoids, which have been shown to have antioxidant and antihypertensive effects in animal and human studies. Several clinical trials have reported that hibiscus supplementation can lead to modest reductions in blood pressure, particularly in individuals with prehypertension or mild hypertension. However, hibiscus supplementation’s optimal dosage and long-term effects on blood pressure control require further investigation.^[2]^ Green tea (Camellia sinensis) is a popular beverage worldwide known for its antioxidant properties and potential health benefits. Green tea contains catechins, particularly epigallocatechin gallate, which have been studied for their effects on cardiovascular health and blood pressure regulation.^[14]^ While some studies have suggested that green tea consumption may have a modest lowering effect on blood pressure, the evidence is inconsistent, and more research is needed to elucidate its potential mechanisms of action and clinical relevance in hypertension management. Olive leaf extract is derived from the leaves of the olive tree (Olea europaea) and has been traditionally used in Mediterranean cultures for its medicinal properties.^[6]^ Olive leaf extract is rich in polyphenols, including oleuropein and hydroxytyrosol, which have antioxidant, anti-inflammatory, and vasodilatory effects. Some studies have reported that olive leaf extract supplementation can reduce blood pressure and improve endothelial function in individuals with hypertension.^[15]^ However, the evidence is limited, and further research is warranted to confirm these findings and establish optimal dosing regimens. While herbal remedies and supplements may offer potential benefits for managing hypertension, it is essential to exercise caution and consult with healthcare professionals before initiating any new treatment regimen.^[16]^ Herbal products are not regulated in the same way as pharmaceutical drugs, and their safety, efficacy, and quality can vary widely. Moreover, herbal remedies can interact with prescription medications and may have adverse effects or contraindications, particularly in individuals with underlying health conditions or taking multiple medications.^[17]^ Healthcare providers should engage in open and non-judgmental discussions with patients about their use of herbal remedies and supplements, inquire about specific products, and provide evidence-based information to help patients make informed decisions.^[6]^ Patients should be encouraged to disclose all herbal products and supplements to their healthcare providers to minimize the risk of potential interactions and adverse effects. Mind-body practices, such as yoga and meditation, have gained increasing attention in recent years for their potential benefits in promoting physical, mental, and emotional well-being.^[8]^ These practices encompass a variety of techniques and disciplines that integrate the mind and body to facilitate relaxation, stress reduction, and self-awareness. While rooted in ancient traditions and spiritual philosophies, mind-body practices have garnered scientific interest for their therapeutic effects on various health conditions, including hypertension, CVD, anxiety, depression, and chronic pain.^[4]^ From ancient India, yoga is a mind-body practice that combines physical postures (asanas), breathing techniques (pranayama), and meditation to promote physical strength, flexibility, balance, and mental clarity. Numerous studies have demonstrated the beneficial effects of yoga on blood pressure regulation, both in individuals with hypertension and prehypertension.^[4]^ Yoga can lower systolic and DBP levels, reduce resting heart rate, and improve cardiovascular function through calming and stress-reducing effects. The mechanisms underlying yoga’s effects on blood pressure are multifaceted and may involve neuroendocrine, autonomic nervous system, and vascular responses. Regular yoga practice has been associated with decreased SNS activity, increased parasympathetic nervous system activity, and reduced levels of stress hormones such as cortisol and adrenaline.^[10]^ These physiological changes contribute to vasodilation, decreased peripheral resistance, and improved blood flow, lowering blood pressure and enhancing cardiovascular health. In addition to its physiological effects, yoga promotes psychological well-being by fostering mindfulness, self-awareness, and emotional resilience. Mindfulness, a core component of yoga practice, involves non-judgmental awareness of present-moment experiences and thoughts.^[1]^ By cultivating mindfulness through yoga and meditation, individuals learn to respond to stressors more skillfully, reduce rumination and negative self-talk, and enhance coping mechanisms for managing stress and anxiety. Meditation is another mind-body practice extensively studied for its effects on blood pressure regulation and stress reduction. Meditation encompasses various techniques and traditions, including mindfulness, focused attention, loving-kindness, and transcendental meditation.^[17]^ These practices share the goal of promoting relaxation, concentration, and mental clarity through focused attention and non-reactive awareness. Numerous studies have demonstrated the beneficial effects of meditation on blood pressure control, with reductions in both systolic and DBP observed in individuals with hypertension and prehypertension.^[7]^ Meditation has been shown to elicit relaxation responses, decrease SNS activity, and enhance parasympathetic tone, leading to improved cardiovascular function and reduced blood pressure levels. One of the key mechanisms underlying meditation’s effects on blood pressure is stress reduction.^[9]^ Chronic stress is a known risk factor for hypertension and CVD, contributing to increased SNS activation, inflammation, and oxidative stress. By inducing a state of deep relaxation and mental calmness, meditation helps counteract the physiological effects of stress, promote emotional balance, and enhance resilience to stressors. Moreover, meditation has been shown to benefit other cardiovascular risk factors, such as insulin resistance, inflammation, and lipid profiles.^[10]^ Regular meditation may improve glycemic control, reduce markers of inflammation, and lower levels of total cholesterol, LDL cholesterol, and triglycerides, thereby reducing the overall risk of cardiovascular events and complications. Integrating mind-body practices into hypertension management offers a holistic, patient-centered approach that complements conventional medical treatments.^[4]^ While pharmacological interventions remain essential for blood pressure control, mind-body practices can serve as valuable adjunctive therapies to enhance overall well-being and quality of life. Healthcare providers should encourage patients with hypertension to explore mind-body practices as part of a comprehensive treatment plan tailored to individual preferences, needs, and abilities.^[8]^ Acupuncture and other alternative therapies have garnered increasing interest as complementary approaches to conventional medical treatments for various health conditions, including hypertension. These alternative therapies encompass various modalities, including acupuncture, acupressure, Traditional Chinese Medicine (TCM), Ayurveda, chiropractic care, massage therapy, and herbal remedies.^[1,7]^ While the evidence supporting the efficacy of these alternative therapies in hypertension management is mixed and often limited, many individuals seek these treatments for their potential benefits in promoting relaxation, reducing stress, and enhancing overall well-being. Acupuncture, originating from ancient China, is a traditional healing practice that involves the insertion of thin needles into specific points on the body to stimulate energy flow and restore balance to the body’s systems.^[10]^ According to TCM principles, hypertension is believed to result from imbalances in the flow of qi (vital energy) and blood within the body. Acupuncture treatments aim to regulate these imbalances by targeting specific acupuncture points associated with blood pressure regulation, stress reduction, and relaxation. While the exact mechanisms underlying acupuncture’s effects on blood pressure remain unclear, several hypotheses have been proposed.^[11]^ Acupuncture may stimulate the release of endorphins and other neurotransmitters, modulate autonomic nervous system activity, promote vasodilation, and reduce inflammation, leading to blood pressure reductions and improved cardiovascular function. Clinical studies investigating the efficacy of acupuncture in hypertension management have yielded mixed results, with some trials reporting modest reductions in blood pressure levels compared to sham or control interventions.^[12]^ Acupressure, a noninvasive technique similar to acupuncture, involves the application of pressure to specific acupuncture points on the body using fingers, thumbs, or specialized tools. Acupressure promotes relaxation, alleviates tension, and enhances circulation, potentially benefiting individuals with hypertension. While limited research has been conducted on acupressure specifically for hypertension, some studies have suggested that acupressure interventions may lead to reductions in blood pressure and perceived stress levels in certain populations. In addition to acupuncture and acupressure, other alternative therapies, such as TCM and Ayurveda, offer holistic approaches to health and wellness that may complement conventional treatments for hypertension.^[13]^ TCM encompasses a range of modalities, including herbal medicine, dietary therapy, qi gong, and tai chi, which aim to restore harmony and balance within the body. Ayurveda, originating from ancient India, emphasizes the interconnectedness of mind, body, and spirit and utilizes practices such as herbal remedies, meditation, yoga, and massage to promote overall well-being.^[14]^ Chiropractic care, massage therapy, and relaxation techniques are also commonly used alternative therapies that may benefit individuals with hypertension by reducing muscle tension, promoting relaxation, and relieving stress. Chiropractic adjustments focus on realigning the spine and musculoskeletal system to improve nerve function and alleviate tension. At the same time, massage therapy involves manual manipulation of soft tissues to enhance circulation and induce relaxation.^[15]^ Relaxation techniques such as deep breathing, guided imagery, and progressive muscle relaxation can help individuals manage stress and lower blood pressure levels. While many individuals turn to alternative therapies for hypertension management, it is essential to approach these treatments with caution and critical evaluation.^[16]^ The evidence supporting the efficacy of alternative therapies for hypertension is often mixed and inconclusive, with limited high-quality research available. Moreover, alternative therapies should not be viewed as substitutes for conventional medical treatments or lifestyle modifications recommended for hypertension management.^[17]^ Healthcare providers should engage in open and non-judgmental discussions with patients about their use of alternative therapies, inquire about specific modalities, and provide evidence-based information to help patients make informed decisions. Patients should be encouraged to disclose all alternative therapies they are pursuing to their healthcare providers to ensure comprehensive and coordinated care.^[18]^ Integrating alternative therapies into hypertension management requires a collaborative approach that considers individual patient preferences, needs, and goals while prioritizing safety, efficacy, and evidence-based practice.^[19]^

## 9. Special considerations

### 9.1. Hypertension in elderly

Hypertension, commonly referred to as high blood pressure, is a significant public health concern that affects individuals of all ages. However, certain populations, such as the elderly, face unique challenges and considerations in the diagnosis, management, and treatment of hypertension.^[20]^ As individuals age, the prevalence of hypertension increases, along with the risk of associated complications, including CVD, stroke, and kidney disease. Understanding the specific characteristics and management strategies for hypertension in the elderly population is essential for optimizing health outcomes and quality of life. One of the defining features of hypertension in the elderly is its high prevalence and increased incidence with advancing age.^[21]^ The aging process is associated with physiological changes that contribute to the development and progression of hypertension, including arterial stiffness, reduced vascular compliance, increased peripheral vascular resistance, and alterations in baroreceptor sensitivity.^[22]^

Additionally, age-related changes in renal function, such as decreased glomerular filtration rate and impaired sodium handling, can further exacerbate blood pressure dysregulation in the elderly. Moreover, the presence of comorbid conditions and age-related changes in organ function can complicate the diagnosis and management of hypertension in the elderly.^[23]^ Common comorbidities in the elderly population, such as diabetes, CKD, and CVD, often coexist with hypertension and may require tailored treatment approaches to address multiple risk factors simultaneously. Furthermore, polypharmacy, or the use of various medications, is prevalent among elderly individuals and can increase the risk of drug interactions, adverse effects, and medication non-adherence.^[23]^ Given the complexity of hypertension management in the elderly, healthcare providers must adopt a comprehensive and individualized approach to treatment. The overarching goal of hypertension management in the elderly is to achieve optimal blood pressure control while minimizing the risk of adverse effects and maintaining overall health and well-being.^[24]^ Evidence-based guidelines, patient preferences, functional status, and frailty considerations should guide treatment decisions. Blood pressure targets for the elderly may differ from those for younger adults and vary depending on individual patient characteristics, comorbid conditions, and cardiovascular risk factors.^[25]^ While lower blood pressure targets (<130/80 mm Hg) have been recommended for certain high-risk elderly populations, such as those with diabetes or CKD, more lenient targets (<140/90 mm Hg) may be appropriate for healthier, low-risk elderly individuals.^[26]^ Lifestyle modifications, including dietary changes, regular physical activity, weight management, smoking cessation, and moderation of alcohol intake, remain the cornerstone of hypertension management in the elderly. However, healthcare providers should consider the feasibility and practicality of lifestyle interventions in the context of an individual’s functional status, cognitive abilities, and social support network.^[27]^ Pharmacological interventions are often necessary to achieve target blood pressure levels in elderly individuals with hypertension, particularly those at higher cardiovascular risk.^[28]^ Antihypertensive medications, including diuretics, CCBs, ACE inhibitors, ARBs, and beta-blockers, are commonly prescribed for elderly patients based on their efficacy, safety profile, and potential for cardiovascular protection.^[29]^ When prescribing antihypertensive medications for elderly patients, healthcare providers should consider factors such as medication tolerability, potential drug interactions, adverse effects, and renal function. Certain antihypertensive medications, such as diuretics and ACE inhibitors, may be preferred in elderly individuals due to their proven efficacy, favorable cardiovascular outcomes, and renoprotective effects.^[30]^ In addition to pharmacological interventions, healthcare providers should prioritize patient education, monitoring, and regular follow-up to ensure optimal blood pressure control and medication adherence in the elderly. Patient-centered care involves engaging elderly patients in shared decision-making, addressing their concerns and preferences, and empowering them to participate actively in their hypertension management.^[31]^

### 9.2. Hypertension in pregnant women

Hypertension in pregnant women, including gestational hypertension, preeclampsia, and chronic hypertension with superimposed preeclampsia, poses significant risks to both maternal and fetal health.^[32]^ Pregnancy-induced hypertension is one of the most common medical complications of pregnancy, affecting approximately 10% of pregnancies worldwide.^[33]^ It is characterized by elevated blood pressure levels that develop after 20 weeks of gestation and typically resolve after delivery. However, if left untreated or poorly managed, hypertension in pregnancy can lead to serious complications, including preterm birth, intrauterine growth restriction, placental abruption, maternal organ damage, and even maternal and fetal death.^[34]^ Gestational hypertension is defined as new-onset hypertension (SBP ≥ 140 mm Hg or DBP ≥ 90 mm Hg) that occurs after 20 weeks of gestation in previously normotensive women. It is often diagnosed based on repeated blood pressure measurements during prenatal visits.^[35]^ Gestational hypertension is typically mild and may not be associated with proteinuria or other signs of organ damage. However, it requires close monitoring to detect signs of progression to preeclampsia, a more severe form of pregnancy-induced hypertension.^[2]^ Preeclampsia is a multisystem disorder characterized by hypertension, proteinuria, and often end-organ dysfunction, such as renal insufficiency, liver involvement, neurological symptoms, or hematological abnormalities. It typically develops after 20 weeks of gestation and can present with a wide range of signs and symptoms, including headache, visual disturbances, epigastric pain, and edema. Preeclampsia is a leading cause of maternal and perinatal morbidity and mortality worldwide, accounting for a significant proportion of maternal deaths and preterm births.^[15]^ Chronic hypertension refers to preexisting hypertension that predates pregnancy or is diagnosed before 20 weeks of gestation. Women with chronic hypertension are at increased risk of developing complications such as preeclampsia, gestational diabetes, preterm birth, and fetal growth restriction. Additionally, women with chronic hypertension are more likely to have comorbidities such as obesity, diabetes, and renal disease, which further increase the risk of adverse pregnancy outcomes.^[6]^ The pathophysiology of hypertension in pregnancy is complex and not fully understood. It is thought to involve abnormalities in placental development, vascular dysfunction, immune dysregulation, and oxidative stress, leading to endothelial damage, inflammation, and impaired perfusion of maternal organs and the placenta. Factors contributing to the development of hypertension in pregnancy include genetic predisposition, maternal age, obesity, insulin resistance, vascular disease, and underlying renal or autoimmune disorders.^[7]^ The management of hypertension in pregnancy aims to reduce maternal and fetal morbidity and mortality by controlling blood pressure, preventing complications, and optimizing maternal and fetal well-being. Treatment strategies may vary depending on the severity of hypertension, gestational age, presence of comorbidities, and fetal status.^[8]^ For women with mild gestational hypertension, close monitoring of blood pressure, fetal growth, and maternal symptoms is typically recommended. Lifestyle modifications, such as dietary changes, reduced salt intake, rest, and regular prenatal care, may be advised to promote maternal well-being and minimize the risk of complications.^[9]^ Women with severe gestational hypertension or signs of preeclampsia may require hospitalization for closer monitoring and initiation of antihypertensive therapy. Antihypertensive medications, such as labetalol, methyldopa, nifedipine, or hydralazine, may be prescribed to lower blood pressure and prevent maternal and fetal complications.^[10]^ The choice of antihypertensive medication depends on factors such as maternal response, fetal safety, and medication availability. Close monitoring of maternal blood pressure, urine protein levels, fetal well-being, and maternal symptoms is essential to assess treatment efficacy and adjust therapy. In cases of severe or refractory hypertension, preeclampsia with severe features, or fetal compromise, delivery may be indicated to mitigate the risk of maternal and fetal complications.^[11]^ The timing and mode of delivery depend on factors such as gestational age, fetal viability, maternal condition, and obstetric considerations. Early delivery may be necessary in cases of severe preeclampsia, eclampsia, hemolysis, elevated liver enzymes, low platelet count syndrome, or fetal distress.^[3]^ Postpartum management of hypertension in pregnancy involves continued monitoring of blood pressure, assessment of maternal symptoms, and surveillance for complications such as postpartum preeclampsia or eclampsia. Women with chronic hypertension may require ongoing monitoring and management of blood pressure in the postpartum period to prevent long-term cardiovascular complications.^[5]^

### 9.3. Hypertension in diabetes

Managing hypertension alongside other health conditions, such as diabetes, presents unique challenges and considerations that require a comprehensive and multidisciplinary approach to optimize patient outcomes.^[7]^ Hypertension and diabetes frequently coexist and share common risk factors, such as obesity, a sedentary lifestyle, and unhealthy dietary habits. The presence of both conditions, known as hypertensive diabetes or diabetic hypertension, significantly increases the risk of cardiovascular complications, including heart disease, stroke, kidney disease, and PVD.^[8]^ Therefore, effective management of hypertension in individuals with diabetes is essential to reduce the risk of cardiovascular events and improve long-term prognosis. The relationship between hypertension and diabetes is bidirectional, with each condition exacerbating the other and contributing to a vicious cycle of cardiovascular risk.^[9]^ Hypertension is a well-established risk factor for the development and progression of diabetes, as elevated blood pressure levels can impair insulin sensitivity, promote insulin resistance, and disrupt glucose metabolism.^[5]^

Conversely, diabetes, particularly type 2 diabetes, is associated with an increased risk of hypertension due to insulin resistance, hyperglycemia, inflammation, oxidative stress, and endothelial dysfunction. Furthermore, the presence of hypertension in individuals with diabetes accelerates the progression of microvascular and macrovascular complications, leading to increased morbidity and mortality.^[6]^ The management of hypertension in individuals with diabetes requires a multifaceted approach that addresses both blood pressure control and glycemic management to reduce cardiovascular risk and improve overall health outcomes.^[7]^ Lifestyle modifications, including dietary changes, regular physical activity, weight management, smoking cessation, and moderation of alcohol intake, form the foundation of hypertension management in individuals with diabetes.^[8]^ These lifestyle interventions lower blood pressure and improve insulin sensitivity, glycemic control, lipid profiles, and cardiovascular health. In addition to lifestyle modifications, pharmacological interventions are often necessary to achieve target blood pressure levels in individuals with diabetes.^[9]^ Antihypertensive medications, such as ACE inhibitors, ARBs, diuretics, CCBs, and beta-blockers, are commonly prescribed to lower blood pressure and reduce cardiovascular risk in individuals with diabetes. The choice of antihypertensive medication depends on factors such as blood pressure levels, presence of comorbidities, renal function, and individual patient preferences.^[10]^ Of particular importance in the management of hypertension in individuals with diabetes are renoprotective agents, such as ACE inhibitors and ARBs, which have been shown to slow the progression of diabetic nephropathy and reduce the risk of ESRD. These medications benefit the RAAS, leading to vasodilation, decreased proteinuria, and preservation of renal function.^[12]^ Therefore, renoprotective agents are recommended as first-line therapy for hypertension in individuals with diabetes, especially those with evidence of renal involvement. Blood pressure targets for individuals with diabetes are generally lower than those for the general population, reflecting the higher cardiovascular risk associated with the combination of hypertension and diabetes.^[15]^ Current guidelines recommend a target blood pressure of <130/80 mm Hg for most individuals with diabetes to reduce the risk of cardiovascular events, stroke, and microvascular complications. However, individualized treatment goals may vary depending on age, comorbidities, life expectancy, and treatment tolerance.^[4]^ Regular monitoring of blood pressure, glycemic control, renal function, lipid levels, and other cardiovascular risk factors is essential to assess treatment efficacy and adjust therapy as needed in individuals with diabetes and hypertension. Healthcare providers should prioritize a patient-centered approach that considers individual preferences, needs, and barriers to adherence in managing hypertension alongside diabetes.^[3]^ Patient education, self-management strategies, and shared decision-making are crucial components of empowering individuals with diabetes to take an active role in their health and well-being.

### 9.4. Hypertension in kidney disease

Hypertension and kidney disease often coexist and share a bidirectional relationship, where hypertension can both cause and result from kidney damage. CKD is a common complication of hypertension.^[8]^ Conversely, hypertension is a significant risk factor for the progression of CKD and the development of ESRD. The interplay between hypertension and kidney disease is complex and multifactorial, involving hemodynamic, neurohormonal, inflammatory, and structural changes in the kidneys and cardiovascular system.^[5]^ Hypertension is a well-established risk factor for the development and progression of CKD, as elevated blood pressure levels can lead to glomerular hyperfiltration, increased intraglomerular pressure, and renal microvascular damage over time. Prolonged exposure to hypertension can cause structural and functional changes in the kidneys, such as glomerulosclerosis, tubulointerstitial fibrosis, arteriolosclerosis, and nephron loss, leading to a decline in renal function and the development of CKD.^[2]^ Hypertension-induced renal damage can occur through various mechanisms, including activation of the RAAS, oxidative stress, inflammation, and endothelial dysfunction.

Conversely, CKD is a common cause of secondary hypertension, as impaired renal function can disrupt sodium and water balance, increase extracellular fluid volume, and activate neurohormonal pathways involved in blood pressure regulation.^[1]^ As kidney function declines, the kidneys’ ability to excrete sodium and regulate blood pressure becomes compromised, leading to sodium retention, volume expansion, and hypertension.^[3]^ Additionally, CKD is associated with alterations in RAAS activity, SNS activation, and vascular tone regulation, further contributing to hypertension development and progression. The presence of hypertension in individuals with CKD accelerates the progression of kidney damage and increases the risk of adverse renal and cardiovascular outcomes. Hypertension exacerbates proteinuria, promotes glomerular injury, and impairs renal autoregulation, leading to further deterioration of kidney function over time.^[19]^

Moreover, hypertension in CKD is a potent risk factor for cardiovascular events, such as myocardial infarction, stroke, heart failure, and sudden cardiac death, which are leading causes of morbidity and mortality in individuals with kidney disease. The management of hypertension in individuals with CKD aims to preserve renal function, reduce proteinuria, prevent cardiovascular events, and improve overall health outcomes.^[20]^ Lifestyle modifications, including dietary changes, weight management, regular physical activity, smoking cessation, and moderation of alcohol intake, are fundamental components of hypertension management in individuals with CKD. These lifestyle interventions lower blood pressure, mitigate cardiovascular risk factors, improve metabolic parameters, and enhance renal and cardiovascular health.^[21]^ Pharmacological interventions are often necessary to achieve target blood pressure levels and prevent the progression of kidney disease in individuals with CKD. Antihypertensive medications, such as ACE inhibitors, ARBs, diuretics, CCBs, and beta-blockers, are commonly prescribed to lower blood pressure and provide renal protection in individuals with CKD.^[22]^ RAAS inhibitors, including ACE inhibitors and ARBs, are particularly beneficial in CKD management due to their renoprotective effects, including reducing proteinuria, slowing the decline in renal function, and delaying the progression to ESRD.^[23]^ In addition to blood pressure control, the management of hypertension in individuals with CKD involves the optimization of other cardiovascular risk factors, such as dyslipidemia, diabetes, and smoking, to reduce the overall burden of CVD.^[24]^ Multidisciplinary care involving nephrologists, cardiologists, primary care providers, dietitians, and pharmacists is essential to provide comprehensive management and support for individuals with hypertension and CKD. Regular monitoring of blood pressure, renal function, electrolytes, and medication adherence is crucial to assess treatment efficacy and adjust therapy as needed.^[25]^

## 10. Monitoring and follow-up

Regular blood pressure monitoring is essential for the effective management of hypertension and the prevention of cardiovascular complications. Healthcare providers rely on accurate blood pressure measurements to assess treatment effectiveness, adjust medication regimens, and guide patient management strategies.^[26]^ Additionally, educating patients on self-monitoring empowers them to take an active role in hypertension management and fosters a collaborative approach between patients and healthcare providers. Regular blood pressure checks cannot be overstated in hypertension management. Blood pressure is a dynamic and fluctuating parameter influenced by various factors such as stress, physical activity, diet, and medication adherence.^[27]^ Therefore, periodic monitoring allows healthcare providers to obtain a comprehensive picture of patients’ blood pressure trends and assess their response to treatment interventions. Adjustments to treatment plans based on monitoring results are critical to hypertension management. Blood pressure targets may vary depending on patient characteristics, comorbid conditions, and cardiovascular risk factors.^[28]^ Regular monitoring enables healthcare providers to evaluate whether patients achieve their blood pressure goals and make timely adjustments to treatment regimens as needed. For instance, if a patient’s blood pressure remains elevated despite adherence to lifestyle modifications and antihypertensive medications, healthcare providers may consider intensifying pharmacological therapy by increasing medication dosages or adding additional agents from different drug classes.^[29]^

Conversely, suppose a patient’s blood pressure is well-controlled on current treatment. In that case, healthcare providers may choose to maintain the existing regimen or even consider reducing medication dosages to minimize the risk of adverse effects. Educating patients on self-monitoring empowers them to participate actively in their hypertension management and fosters a sense of ownership over their health.^[30]^ Self-monitoring allows patients to track their blood pressure readings at home using portable devices and transmit this information to their healthcare providers for review and interpretation. By regularly monitoring their blood pressure at home, patients can detect any fluctuations or trends that may require attention and facilitate early intervention.

Furthermore, self-monitoring enables patients to identify potential triggers for elevated blood pressure, such as stress, dietary factors, or medication non-adherence, and make lifestyle adjustments accordingly.^[14]^ For example, suppose a patient notices that their blood pressure readings are consistently higher in the morning. In that case, they may be prompted to practice relaxation techniques or modify their morning routine to reduce stress.^[4]^ In addition to self-monitoring, patient education should also encompass instruction on proper blood pressure measurement techniques, including selecting appropriate cuff sizes, positioning the arm at the heart level, and avoiding factors that can artificially elevate blood pressure readings, such as caffeine consumption or smoking. Healthcare providers should also emphasize the importance of regular follow-up visits to review blood pressure trends, assess treatment adherence, and address any concerns or questions that patients may have.^[6]^

## 11. Future directions and research

One area of ongoing research involves the development of novel pharmacological agents and treatment strategies for hypertension. Despite the availability of numerous antihypertensive medications, there remains a need for more effective and better-tolerated therapies, particularly for individuals with resistant hypertension or those who experience adverse effects from existing treatments.^[16]^ Researchers are exploring innovative drug targets, such as novel mechanisms of action within the RAAS, endothelial function pathways, SNS modulation, and sodium-glucose cotransporter 2 inhibition, to identify new therapeutic options for hypertension management.^[17]^ In recent years, there has been growing interest in precision medicine approaches to hypertension management, which aim to tailor treatment strategies to individual patient characteristics, including genetic factors, biomarkers, comorbidities, and lifestyle factors. Advances in genomic research have identified genetic variants associated with blood pressure regulation and response to antihypertensive medications, offering potential insights into personalized treatment approaches.^[18]^

Similarly, biomarkers, such as circulating markers of inflammation, oxidative stress, and endothelial dysfunction, may help identify high-risk individuals and guide targeted interventions to prevent hypertension-related complications. Technological innovations are crucial in diagnosing, monitoring, and managing hypertension. Wearable devices, such as ambulatory blood pressure monitors, smartwatches, and remote monitoring systems, enable continuous tracking of blood pressure and other cardiovascular parameters outside of the clinical setting, providing valuable data for personalized risk assessment and treatment optimization.^[19]^ Telemedicine and digital health platforms offer remote patient monitoring, virtual consultations, and lifestyle coaching opportunities, enhancing access to care and improving patient engagement in hypertension management.^[20]^ Another area of active research is the role of lifestyle interventions and non-pharmacological therapies in hypertension prevention and management.^[20]^ Lifestyle factors, including diet, physical activity, stress management, and sleep quality, significantly impact blood pressure regulation and cardiovascular health. Emerging evidence suggests that comprehensive lifestyle interventions, such as the DASH diet, mindfulness-based stress reduction, aerobic exercise, and sleep hygiene practices, can effectively lower blood pressure, reduce cardiovascular risk, and complement pharmacological treatments in hypertension management.^[21]^ In addition to exploring new treatments and technologies, there are several areas for further investigation in hypertension research. One area of interest is understanding the long-term cardiovascular effects of emerging therapies, particularly those targeting novel pathways or mechanisms of action.^[22]^ Longitudinal studies and clinical trials are needed to assess the safety, efficacy, and cardiovascular outcomes of new antihypertensive medications and treatment strategies, especially in diverse patient populations and real-world settings.

Furthermore, there is a need for research focused on addressing disparities in hypertension prevalence, awareness, treatment, and control among different demographic groups, including racial and ethnic minorities, socioeconomically disadvantaged populations, and individuals with limited access to healthcare resources.^[23]^ Identifying barriers to hypertension management and implementing culturally tailored interventions and community-based programs are essential to reducing disparities and improving cardiovascular health equity.^[24]^ Additionally, future research should explore the impact of environmental factors, such as air pollution, noise exposure, and neighborhood characteristics, on blood pressure regulation and hypertension risk. Understanding the complex interplay between environmental determinants and individual susceptibility to hypertension may inform public health policies and urban planning initiatives to create healthier built environments and reduce CVD burden.^[36]^

## 12. Conclusion

The management of hypertension requires a multifaceted approach that addresses lifestyle modifications, pharmacological interventions, and regular monitoring to reduce cardiovascular risk and improve patient outcomes. Healthcare providers can effectively guide patients in achieving optimal blood pressure control and preventing complications associated with this chronic condition through a comprehensive understanding of the pathophysiology, risk factors, and treatment options for hypertension. By implementing evidence-based strategies, including dietary changes, regular exercise, stress management techniques, and medication adherence, individuals with hypertension can take proactive steps to manage their blood pressure and improve their overall health and well-being.

## 13. Call to action

As healthcare professionals, we must prioritize hypertension management as a public health priority and advocate for greater awareness, screening, and treatment of this prevalent condition. We must continue to educate patients, families, and communities about the importance of blood pressure control and empower them with the knowledge and resources needed to make informed decisions about their health. Additionally, we must advocate for policies and initiatives that promote access to affordable healthcare, healthy food options, and safe environments conducive to physical activity. Together, through collaborative efforts and collective action, we can significantly reduce the burden of hypertension and improve cardiovascular health for all individuals.

## Acknowledgments

The authors would like to express gratitude to all individuals and institutions that contributed to the completion of this paper. Their support, guidance, and encouragement throughout the research process are deeply appreciated.

## Author contributions

**Conceptualization:** Chukwuka Elendu, Dependable C. Amaechi, Tochi C. Elendu, Emmanuel C. Amaechi, Ijeoma D. Elendu.

**Data curation:** Chukwuka Elendu, Dependable C. Amaechi, Tochi C. Elendu, Emmanuel C. Amaechi, Ijeoma D. Elendu.

**Formal analysis:** Chukwuka Elendu, Dependable C. Amaechi, Tochi C. Elendu, Emmanuel C. Amaechi, Ijeoma D. Elendu.

**Funding acquisition:** Chukwuka Elendu, Dependable C. Amaechi, Tochi C. Elendu, Emmanuel C. Amaechi, Ijeoma D. Elendu.

**Investigation:** Chukwuka Elendu, Dependable C. Amaechi, Tochi C. Elendu, Emmanuel C. Amaechi, Ijeoma D. Elendu.

**Methodology:** Chukwuka Elendu, Dependable C. Amaechi, Tochi C. Elendu, Emmanuel C. Amaechi, Ijeoma D. Elendu.

**Project administration:** Chukwuka Elendu, Dependable C. Amaechi, Tochi C. Elendu, Emmanuel C. Amaechi, Ijeoma D. Elendu.

**Resources:** Chukwuka Elendu, Dependable C. Amaechi, Tochi C. Elendu, Emmanuel C. Amaechi, Ijeoma D. Elendu.

**Software:** Chukwuka Elendu, Dependable C. Amaechi, Tochi C. Elendu, Emmanuel C. Amaechi, Ijeoma D. Elendu.

**Supervision:** Chukwuka Elendu, Dependable C. Amaechi, Tochi C. Elendu, Emmanuel C. Amaechi, Ijeoma D. Elendu.

**Validation:** Chukwuka Elendu, Dependable C. Amaechi, Tochi C. Elendu, Emmanuel C. Amaechi, Ijeoma D. Elendu.

**Visualization:** Chukwuka Elendu, Dependable C. Amaechi, Tochi C. Elendu, Emmanuel C. Amaechi, Ijeoma D. Elendu.

**Writing – original draft:** Chukwuka Elendu, Dependable C. Amaechi, Tochi C. Elendu, Emmanuel C. Amaechi, Ijeoma D. Elendu.

**Writing – review & editing:** Chukwuka Elendu, Dependable C. Amaechi, Tochi C. Elendu, Emmanuel C. Amaechi, Ijeoma D. Elendu.
